# Renal PIEZO2 is an essential regulator of renin

**DOI:** 10.1016/j.cell.2025.11.013

**Published:** 2025-12-04

**Authors:** Rose Z. Hill, Jonathan W. Nelson, Georgina Gyarmati, Silvia Medrano, Sepenta Shirvan, James A. McCormick, Sebastian Burquez, Jeanine Ahmed, Diana G. Eng, Jan Wysocki, Adrienne E. Dubin, M. Rocio Servin-Vences, Arjun Lakshmanan, R. Ariel Gomez, Maria Luisa S. Sequeira-Lopez, Stuart J. Shankland, Daniel Batlle, Jeffrey H. Miner, Janos Peti-Peterdi, Ardem Patapoutian

**Affiliations:** 1 Howard Hughes Medical Institute, Department of Neuroscience, Dorris Neuroscience Center, The Scripps Research Institute, La Jolla, CA 92037, USA.; 2 Division of Nephrology and Hypertension, Department of Medicine, Keck School of Medicine, University of Southern California, Los Angeles, CA 90033, USA.; 3 Department of Physiology and Neuroscience, Zilkha Neurogenetic Institute, Keck School of Medicine, University of Southern California, Los Angeles, CA 90033, USA.; 4 Department of Pediatrics, University of Virginia School of Medicine, University of Virginia, Charlottesville, VA, 22903, USA; 5 Division of Nephrology and Hypertension, Department of Medicine, Oregon Health & Science University, Portland, OR, 97239 USA.; 6 Division of Nephrology, Department of Medicine, University of Washington; Seattle, WA 98195, USA.; 7 Division of Nephrology and Hypertension, Department of Medicine, Northwestern University Feinberg School of Medicine, Chicago, IL 60611, USA.; 8 Division of Nephrology, Department of Medicine, Washington University School of Medicine, St. Louis, MO 63110, USA.

**Keywords:** renin-angiotensin-aldosterone system, PIEZO2, mechanotransduction, ion channel, kidney, mesangial cell, juxtaglomerular granular cell, filtration, blood volume, renin, blood pressure, glomerulus, calcium

## Abstract

Renin synthesis and release is the rate-limiting step of the renin-angiotensin-aldosterone system (RAAS) that controls fluid homeostasis. A major activator of RAAS is a decrease in perfusion pressure within the kidneys, suggesting a link between renal mechanotransduction and renin. The identity of the mechanosensor(s) in the kidneys and their physiological significance to the RAAS is unclear. We found that loss of the force-gated nonselective cation channel PIEZO2 in cells of renin lineage dysregulated the RAAS by elevating renin. We observed that PIEZO2 is expressed in renin-producing juxtaglomerular granular cells and is required for their calcium dynamics *in vivo*. PIEZO2 deficiency in cells of renin lineage drives renin- and MAS Receptor-dependent glomerular hyperfiltration and regulates the RAAS during acute and chronic blood volume challenges. Collectively, our study identifies PIEZO2 as an essential regulator of juxtaglomerular granular cell calcium activity and renin *in vivo*.

## eTOC Blurb/In Brief

The force-sensing ion channel PIEZO2 is an essential regulator of the renin-angiotensin-aldosterone system (RAAS), a pathway that controls blood volume. Loss of PIEZO2 in the kidney cells that produce renin disrupts cellular calcium dynamics and levels of the enzyme in mice, linking mechanotransduction in the kidney to the RAAS.

## Graphical Abstract

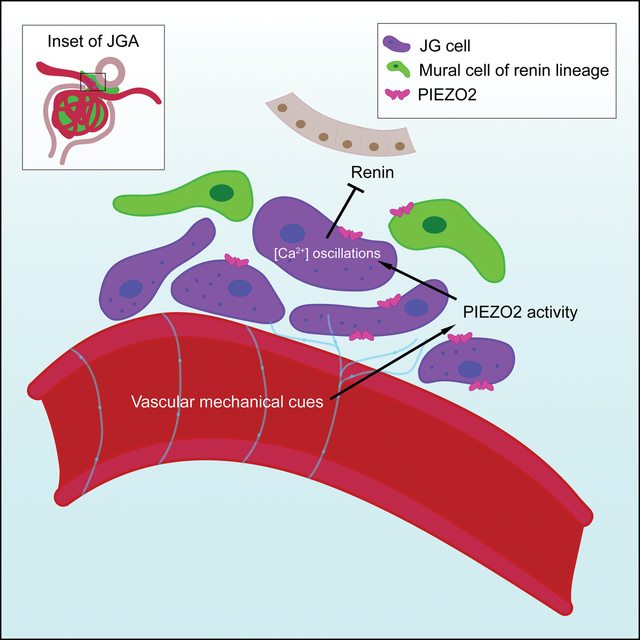

## Introduction

The scientist Homer Smith wrote, “Our kidneys constitute the major foundation of our physiological freedom.”^1^ Vertebrate animals control their blood volume through feedback loops spanning the renal, nervous, and cardiovascular systems. Importantly, these processes allow organisms to adapt to ever-changing and physiologically demanding environments. The sensory cells that detect changes in blood volume and/or pressure are called baroreceptors. Neuronal baroreceptors innervating the aortic arch utilize PIEZO ion channel-dependent mechanotransduction to monitor vascular mechanical forces. The activation of these baroreceptors rapidly alters cardiac function and vascular resistance via the autonomic nervous system.^2–4^ A non-neuronal baroreceptor in the kidney responds to volume depletion through the production and release of the hormone and enzyme renin.^5^ This process forms the rate-limiting steps of the renin-angiotensin-aldosterone system (RAAS), a hormonal cascade that increases vascular tone and conserves electrolytes and water.^5^ Renin is synthesized by and secreted from the juxtaglomerular granular (JG) cells that decorate the terminal afferent arteriole feeding the glomeruli within the kidney.^5–7^ Renin production and/or release from JG cells is stimulated by three main routes within the JG apparatus (JGA): 1) norepinephrine released from sympathetic nerves that activates beta 1 adrenergic G-protein coupled receptors and stimulates renin in a cAMP- and PKA-dependent manner,^8–10^ 2) a reduction in tubular salt levels that triggers macula densa cells to secrete prostaglandins onto the JG cells and activate GPCRs PTGER2 and PTGER4,^11–14^ and, 3) proposed direct mechanosensation that stimulates renin release from JG cells in an inversely calcium-dependent manner in response to arteriolar hemodynamics.^7,15,16^

Of these mechanisms to stimulate renin, the least understood is mechanosensation. Current models propose that mechanical stress, vasomotion, and/or changes in myogenic tone of the afferent arteriole stimulate calcium oscillations and influx in the JG cells to suppress renin. Loss of these signals, such as through volume depletion, would conversely suppress calcium influx to stimulate renin. Several ion channels and pathways are proposed to mediate mechanotransduction in JG cells,^17–20^ yet their role in the RAAS is unclear. JG cell calcium oscillations are observed *in vivo* using 2-photon imaging, but their mechanistic origins remain mysterious.^21^ While it is hypothesized that the JG cells themselves are the mechanosensors,^19^ they are reported to be electrically coupled to adjacent mesangial cells, mural cells, or vascular smooth muscle cells.^20,22–24^ As such, the cellular identity of the mechanotransducer(s) cannot be assumed and the *in vivo* physiological consequences of loss of JG cell mechanosensitivity are unknown. In our present study, we examined the molecular identity, cellular site-of-action, and physiological significance of mechanotransduction in the regulation of renin and the RAAS.

Considering the relationship between intracellular calcium levels and renin, we hypothesized that mechanically activated nonselective cation channels underlie a component of the renal baroreceptor. PIEZO1 and PIEZO2 comprise a family of ion channels that are exclusively gated by mechanical force.^25^ PIEZOs are necessary and sufficient for the ability of cells to detect and respond to physiologically relevant mechanical stimuli; as such, cells expressing PIEZOs display mechanically activated (MA) currents.^25^ PIEZO1 is endogenously expressed in many tissue types including vascular endothelium and smooth muscle where it governs vascular development and function.^26,27^ PIEZO2 is mainly expressed in peripheral sensory neurons and specialized accessory sensory cells where it mediates gentle touch sensation, proprioception, and excretory functions.^28^

## Results

### PIEZO2 is expressed in JG and mesangial cells of the kidney

**Figure 1.**
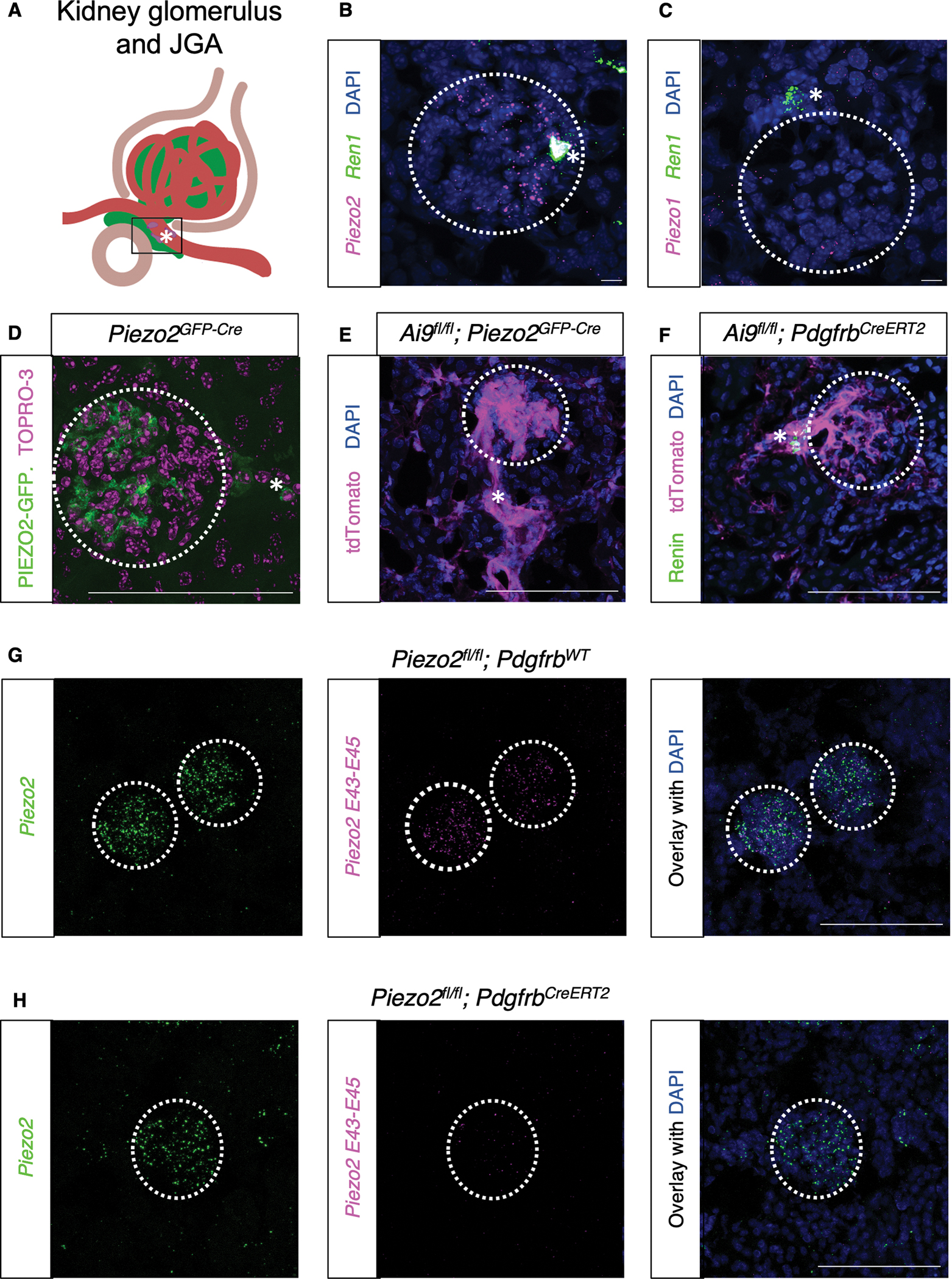
PIEZO2 is expressed in *Pdgfrb*-lineage mesangial and JG cells of the kidney. **A.** Cartoon of a kidney glomerulus and associated JGA (boxed and with white asterisk). **B.** smFISH of sectioned C57BL6/J mouse kidney (scale = 10 μm). **C.** smFISH of sectioned C57BL6/J mouse kidney (scale = 10 μm). **D.** Sectioned mouse kidney. **E.** Sectioned mouse kidney with native tdTomato fluorescence marking PIEZO2 lineage and counterstained with DAPI. **F.** Sectioned mouse kidney with native tdTomato fluorescence, stained with anti-Renin antibody, and counterstained with DAPI. Asterisk (*) indicates extraglomerular expression at putative Renin+ vascular pole. Each experiment was repeated on N=2 mice with two technical replicates (slides) per experimental condition. **G-H**. smFISH of sectioned mouse kidney for *Piezo2* (left), *Piezo2 E43–45* (center), and merged with DAPI (right). Scale bars = 100 μm unless otherwise indicated. Dotted circles indicate renal corpuscles. **G** is representative of 28 glomeruli from two slides each from two mice, where 28 of 28 glomeruli had *Piezo2*^+^ and *Piezo2 E43–45*^+^ cells. **H** is representative of 31 glomeruli from two slides each from two mice, where 31 glomeruli had *Piezo2*^+^ cells and 1 glomerulus had detectable *Piezo2 E43–45*^+^ cells. See also Table S1.

To characterize the expression of MA ion channels in the kidneys, we examined mRNA transcript localization of PIEZO channels using single molecule fluorescence in situ hybridization (smFISH). We found that *Piezo2* and not *Piezo1* was expressed in *Ren1-*expressing putative JG cells and in mesangial cells, aligning with published studies (Figures 1A–C).^29,30^ We did not observe *Piezo2* transcript in other structures within the cortex or medulla. Using a *Piezo2*^*GFP-Cre*^ knock-in mouse,^31^ we observed PIEZO2-GFP fusion protein expression in glomerular mesangial and periglomerular presumptive JG cells (Figure 1D) as well as glomerular and JG cell labeling of *Piezo2*-lineage cells (Figure 1E).^32^ To investigate whether *Piezo2* expression was restricted to stromal cells including glomerular and JG cells, we turned to an inducible Cre recombinase mouse line, *Pdgfrb*^*CreERT2*^, that selectively targets stromal cells including mesangial cells, renin-expressing JG cells, and mural cells of renin lineage after tamoxifen administration to adult mice (Figure 1F, Table S1).^33^ We found that tamoxifen profoundly ablated *Piezo2* expression in the kidneys of *Pdgfrb*^*CreERT2*^ but not *Pdgfrb*^*WT*^ animals through smFISH targeting the *loxp*-flanked exons^34^ (*Piezo2 E43-E45*) and the entire transcript (*Piezo2,* Figures 1G–H).

### snRNA-seq reveals *Piezo2* expression within three distinct kidney stromal cell types

**Figure 2.**
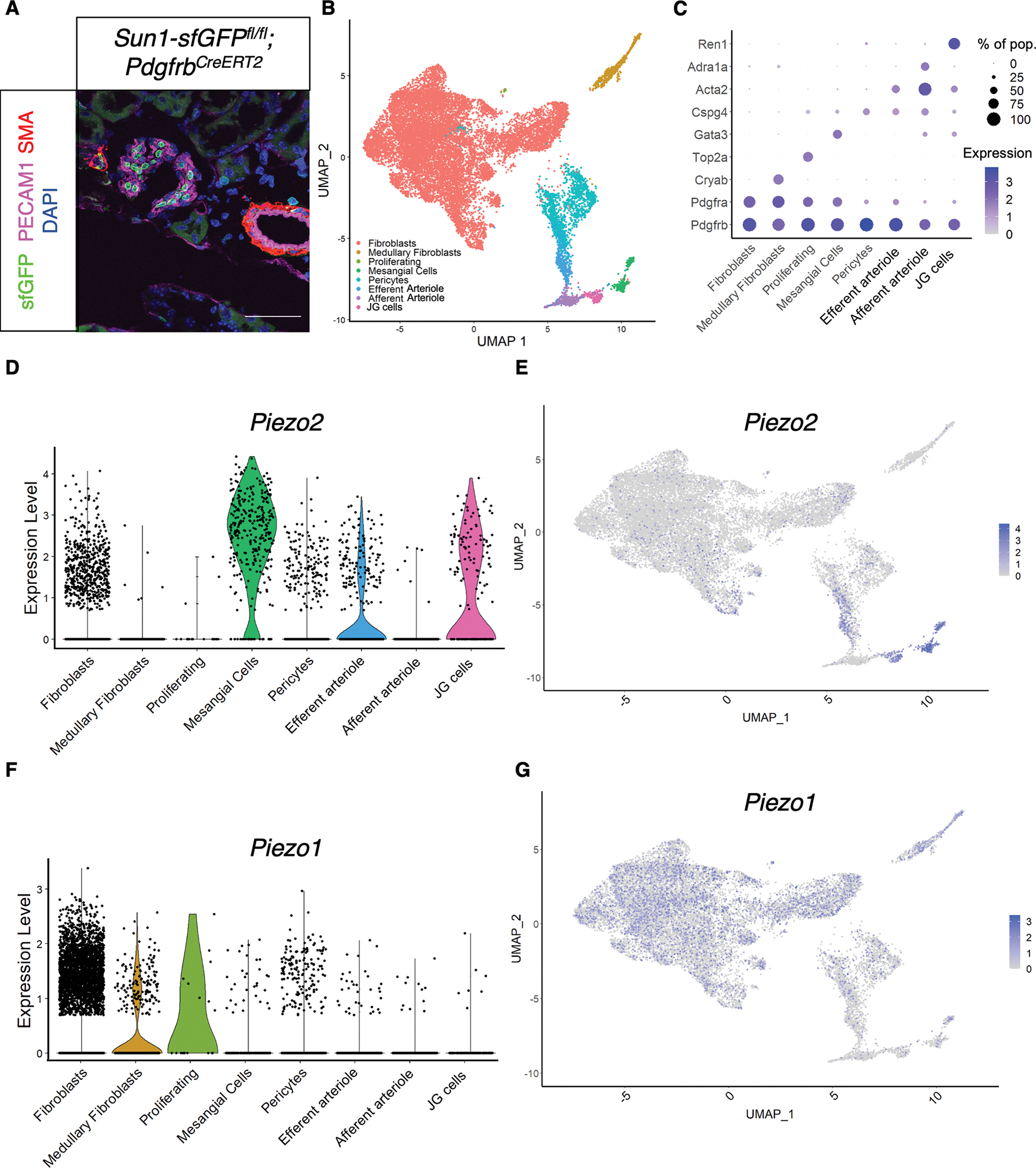
INTACT snRNA-seq supports selective expression of *Piezo2* and not *Piezo1* in mesangial, JG cells, and efferent arteriole cells of the mouse kidney. **A.** Representative *Sun1-sfGFP*^*fl/fl*^*; Pdgfrb*^*CreERT2*^ mouse kidney. **B.** UMAP projection of *Sun1-sfGFP*^*fl/fl*^*; Pdgfrb*^*CreERT2*^ snRNA-seq. **C**. Multidimensional dot plot of cell type-specific markers used to identify clusters corresponding to distinct *Pdgfrb*^+^ kidney cell populations in **D**. Violin plot of *Piezo2*. **E.** Feature plot of *Piezo2* expression in UMAP space. **F**. Violin plot of *Piezo1*. **G.** Feature plot of *Piezo1* expression in UMAP space. See also Figures S1–S3.

Stromal cells are broadly targeted by the *Pdgfrb*^*CreERT2*^ mouse line.^33,35,36^ As our smFISH experiments suggest *Piezo2* expression within the kidney is restricted to a subset of stromal cells associated with or near the glomeruli, we used a comprehensive snRNA-seq approach^37^ to assess stromal *Piezo* gene expression in the kidneys. We generated *Sun1-sfGFP*^*fl/fl*^*; Pdgfrb*^*CreERT2*^ mice that inducibly express nuclear-localized sfGFP^38,39^ (Figure 2A, Table S1). We sequenced 17,990 GFP^+^ kidney nuclei with a median read depth of 2,855 unique molecular identifiers and a median detection of 1,555 genes (Figures S1A–E). We generated a 2D reduction using uniform manifold approximation and projection (UMAP). We categorized captured nuclei into eight clusters based upon marker gene(s): fibroblasts^40^ (*Pdgfra*^+^), medullary fibroblasts^40^ (*Pdgfra*^+^, *Cryab*^+^), proliferating cells (*Top2a*^+^), mesangial cells^35^ (*Gata3*^+^), pericytes (*Cspg4*^+^), efferent arteriole^41^ (*Acta2*^+^), afferent arteriole^41^ (*Acta2*^+^, *Adra1a*^+^), and JG cells^42^ (*Ren1*^+^, Figures 2B–C, S2A–B). *Piezo2* was expressed in mesangial cells and JG cells and enriched in the efferent arteriole population compared to the afferent arteriole population (Figures 2D–E). The arteriolar mural cells are the sites of the myogenic responses that regulate the glomerular filtration rate (GFR) and can take on a renin-expressing identity during challenge.^43^ We additionally validated co-expression of *Piezo2* and not *Piezo1* with *Pdgfrb* by smFISH, with low levels of glomerular *Piezo1* in putative *Pdgfrb*-negative cells consistent with previously reported expression of this channel at low levels in capillary endothelial cells and/or podocytes^44^ (Figures S3A–C).

In concordance with our smFISH data, we did not observe expression of *Piezo1* in any of the *Piezo2*^+^ populations by snRNA-seq. *Piezo1* transcripts were detected in medullary fibroblasts and proliferating cells (Figures 2F–G). The lack of observed co-expression of *Piezo* genes contrasted with a recent study proposing that PIEZO1 contributes to JG cell function.^18^ As confirmation, we performed IHC of PIEZO1-tdTomato fusion protein from a *Piezo1*^*tdTomato*^ knock-in mouse line.^45^ Aligning with previous work,^44^ we observed expression restricted to basal aspects of a subset of tubular epithelial cells (Figure S3D). We additionally performed smFISH of *PIEZO2* on human kidney sections and observed overlap of *PIEZO2* and *PDGFRB* in the kidney cortex (Figure S3E). While further investigation of human kidneys will be needed to more carefully elucidate PIEZO2 expression patterns, our findings suggest that *PIEZO2* is expressed within kidney mural cells and align with snRNA-seq datasets that show expression of *PIEZO2* in mesangial and renin-expressing cells.^46^

### PIEZO2 in mural cells of renin lineage is an essential regulator of plasma renin

We hypothesized that PIEZO2 might play a role in regulating RAAS within JG cells. To identify additional transgenic mouse lines aside from *Pdgfrb*^*CreERT2*^ (Figures 1G–H, S3F–G) that would be useful for investigation the function of PIEZO2 in the kidneys, we assessed Cre lines targeting cells of renin lineage and/or stromal cells. We performed IHC of kidneys from *Ren1c*^*Cre*^ (*Ren*^*Cre*^ )^47,48^ and *FoxD1*^*GFP-Cre* 36^ reporter mouse lines. We observed that *Ren*^*Cre*^ targets mural cells of renin lineage encompassing JG cells and a varying subset of renin-negative renal arteriolar cells, while sparing intraglomerular mesangial cells (Figures S3H–I, Table S1).^47^ We concluded that this genetic tool would enable differentiation of mesangial from JG/arteriolar functions when compared alongside *Pdgfrb*^*CreERT2*^ during *in vivo* experiments. *FoxD1*^*GFP-Cre*^ targeted a broad population of cells encompassing mural cells and fibroblasts during development, while sparing tubules and endothelial cells, as expected (Figures S3J–K, Table S1).^49^ We concluded that this line would be useful for ascertaining developmental roles of PIEZO2 in the kidney by comparing its results with those from the adult-induced *Pdgfrb*^*CreERT2*^ line. We also investigated whether we could target adult renin-expressing cells (presumptive JG cells). Our initial testing of a inducible *Ren*^*CreER*^ reporter line^47^ showed selective targeting of adult renin-expressing cells; however, we observed only partial targeting of JG cells, rendering it unsuitable for loss-of-function studies (Figure S3L). Our analysis of these lines suggested we could distinguish the functions of PIEZO2 in all PIEZO2^+^ renal cells versus mural cells of renin lineage, as well as developmental versus adult effects by examining *Pdgfrb*^*CreERT2*^, *FoxD1*^*GFP-Cre*^, and *Ren*^*Cre*^ conditional knockouts in combination. When considering extrarenal Cre activity, it is worth noting that PIEZO2, unlike PIEZO1, is present in relatively fewer cell types and mainly found within the peripheral nervous system.^28^ Although PIEZO2 expression has been sporadically observed in vascular endothelial cells,^52^ we did not observe this in our smFISH of kidney.

**Figure 3.**
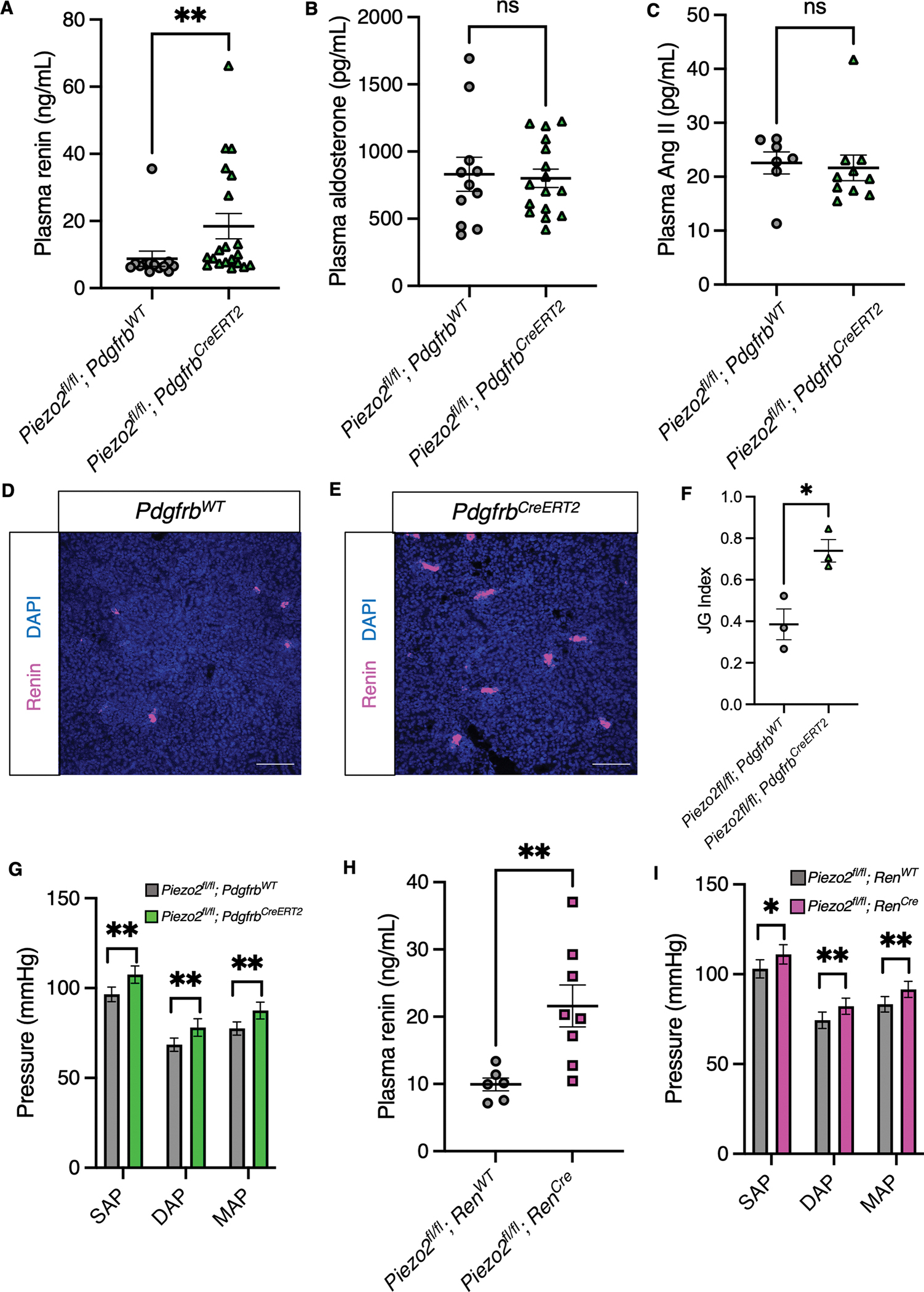
PIEZO2 regulates renin. **A.** Plasma renin levels (Mann–Whitney: **p = 0.0019, U = 48; n = 20 *Pdgfrb*^*WT*^ and 13 *Pdgfrb*^*CreERT2*^ mice). **B.** Plasma aldosterone levels (Mann–Whitney: *p* = 0.8653, U = 84; n = 11 *Pdgfrb*^*WT*^ and 16 *Pdgfrb*^*CreERT2*^ mice). **C.** Plasma Ang II levels (Mann–Whitney: *p* = 0.1932, U = 21; n = 7 *Pdgfrb*^*WT*^ and 10 *Pdgfrb*^*CreERT2*^ mice). **D.** Sectioned kidney representative of n = 4 fields-of-view (two each on two slides) from N = 3 mice from *Piezo2*^*fl/fl*^*; Pdgfrb*^*WT*^ mouse (scale = 100 μm). **E.** Sectioned kidney representative of n = 4 fields-of-view (two each on two slides) from N = 3 mice from *Piezo2*^*fl/fl*^*; Pdgfrb*^*CreERT2*^ mouse (scale = 100 μm). **F.** JG index (Welch’s t-test: *p = 0.0215, t = 3.847; df = 3.673; N = 3 *Pdgfrb*^*WT*^ and 3 *Pdgfrb*^*CreERT2*^ kidneys from 3 mice each). **G.** Systemic blood pressure (systolic/SAP; diastolic/DAP; and mean arterial pressure/MAP; two-tailed nested *t*-tests (left to right): **p_*SAP*_ = 0.0013, *t* = 3.899, d.f. = 16; **p_*DAP*_ = 0.0056, *t* = 3.197, d.f. = 16; **p_*MAP*_ = 0.0027, *t* = 3.546, d.f. = 16; n = 10 *Pdgfrb*^*WT*^ and 8 *Pdgfrb*^*CreERT2*^ mice). **H.** Plasma renin levels (Mann–Whitney: **p = 0.0047, U = 3; n = 6 *Ren*^*WT*^ and 8 *Ren*^*Cre*^ mice). **I.** Systemic blood pressure (two-tailed nested *t*-tests (left to right): *p_*SAP*_ = 0.0147, *t* = 2.716, d.f. = 17; **p_*DAP*_ = 0.0097, *t* = 2.913, d.f. = 17; **p_*MAP*_ = 0.0060, *t* = 3.137, d.f. = 17; n = 9 *Ren*^*WT*^ and 10 *Ren*^*Cre*^ mice). Each experiment was performed on at least two independent cohorts of mice, and error bars represent mean ± s.e.m. See also Table S1 and Figures S4–S7.

We next investigated the consequences of loss of functional PIEZO2 on renin levels and RAAS components. As a first approach, we measured renin levels in plasma harvested from naïve *Piezo2* conditional knockout mice. Initially, to avoid potential developmental confounds, we examined the inducible *Piezo2*^*fl/fl*^*; Pdgfrb*^*CreERT2*^ conditional knockout mice. We observed a significant increase in plasma renin levels in the *Piezo2*^*fl/fl*^*; Pdgfrb*^*CreERT2*^ mice compared to *Piezo2*^*fl/fl*^*; Pdgfrb*^*WT*^ controls (Figure 3A). Of note, renin levels in the conditional knockout mice were variable, suggesting that other pathways may compensate for the effects of PIEZO2 on renin levels under naïve conditions. This effect was maintained in *Piezo1*^*fl/fl*^*; Piezo2*^*fl/fl*^*; Pdgfrb*^*CreERT2*^ mice lacking both ion channels (Figure S4A), consistent with our observations that PIEZO2 and not PIEZO1 is expressed in our cell types of interest. As expected, *Piezo2*^*fl/fl*^*; FoxD1*^*GFP-Cre*^ animals phenocopied the *Piezo2*^*fl/fl*^*; Pdgfrb*^*CreERT2*^ mice (Figure S4B). Notably, we did not observe elevations in downstream RAAS components angiotensin (Ang) II and aldosterone (Figures 3B–C). This finding was surprising given that renin catalyzes the formation of Ang I from angiotensinogen. We speculated that the expected increase in Ang II might be prevented by enhanced enzymatic processing of the peptide.

We also examined whether renal renin was elevated in addition to circulating renin through IHC in kidneys from wild-type and *Piezo2*^*fl/fl*^*; Pdgfrb*^*CreERT2*^ mice and quantification of the JG index.^50^ Indeed, we observed a significant increase in the JG index, demonstrating that loss of PIEZO2 in mesangial, efferent arteriolar, and JG cells confers elevated renal and circulating renin (Figures 3D–F). Notably, we did not observe substantial recruitment of renin expression in cells along the renal vasculature, suggesting that the renin expression was mainly restricted to JG cells. We measured the blood pressure of *Piezo2*^*fl/fl*^*; Pdgfrb*^*WT*^ and *Pdgfrb*^*CreERT2*^ mice using the volume pressure recording (VPR) method^51,52^ and found a significant difference in *Pdgfrb*^*CreERT2*^ mice that was elevated (Mean MAP ± SD: 77.53 ± 11.59 mmHg vs. 87.54 ± 13.21 mmHg; N=10, 8; **p = 0.0027, two-tailed nested *t-*test) but not indicative of hypertension, with statistically indistinguishable differences in heart rate during the recordings (Figures 3G, S5A–F). While hyperreninemia can drive hypertension through elevated Ang II,^53^ this increase was not observed (Figure 3C). It is possible that the variable and elevated baseline renin observed in the conditional knockout mice contributes to sporadic elevations in Ang II not captured by our single-point measurements, but which are still capable of triggering mildly elevated blood pressure, or that the effect was due to factors independent of the RAAS.

To determine which renal cell type(s) contribute to elevated renin, we measured plasma renin in *Piezo2*^*fl/fl*^*; Ren*^*Cre*^ conditional knockouts and controls. Plasma renin was elevated with loss of PIEZO2 in the cells of renin lineage (Figure 3H). We also observed a mild increase in blood pressure (Figures 3I, S6A–F; Mean MAP ± SD: 83.30 ± 12.96 mmHg vs. 91.53 ± 14.31 mmHg; N=9, 10; **p = 0.0060, two-tailed nested *t-*test). Importantly, plasma renin activity (PRA) was elevated with conditional loss of PIEZO2 (Figure S7A). Moreover, our conditional knockout mice had no difference in plasma electrolytes that could alternatively affect the RAAS (Figures S7B–C). We also observed elevated local renin in the JGA (Figures S7D–F). We conclude that the effects of loss of PIEZO2 on local and circulating renin are not dependent on intraglomerular mesangial cells.^54^ While the most straightforward explanation for our observations implicates renin-expressing JG cells, we cannot rule out an indirect contribution of PIEZO2 in efferent arteriolar mural cells of renin lineage.^55–57^ Therefore, we conclude that PIEZO2 in cells of renin lineage inclusive of JG cells is required for regulation of renin *in vivo.*

### PIEZO2 is required for intracellular calcium oscillations in JG cells

**Figure 4.**
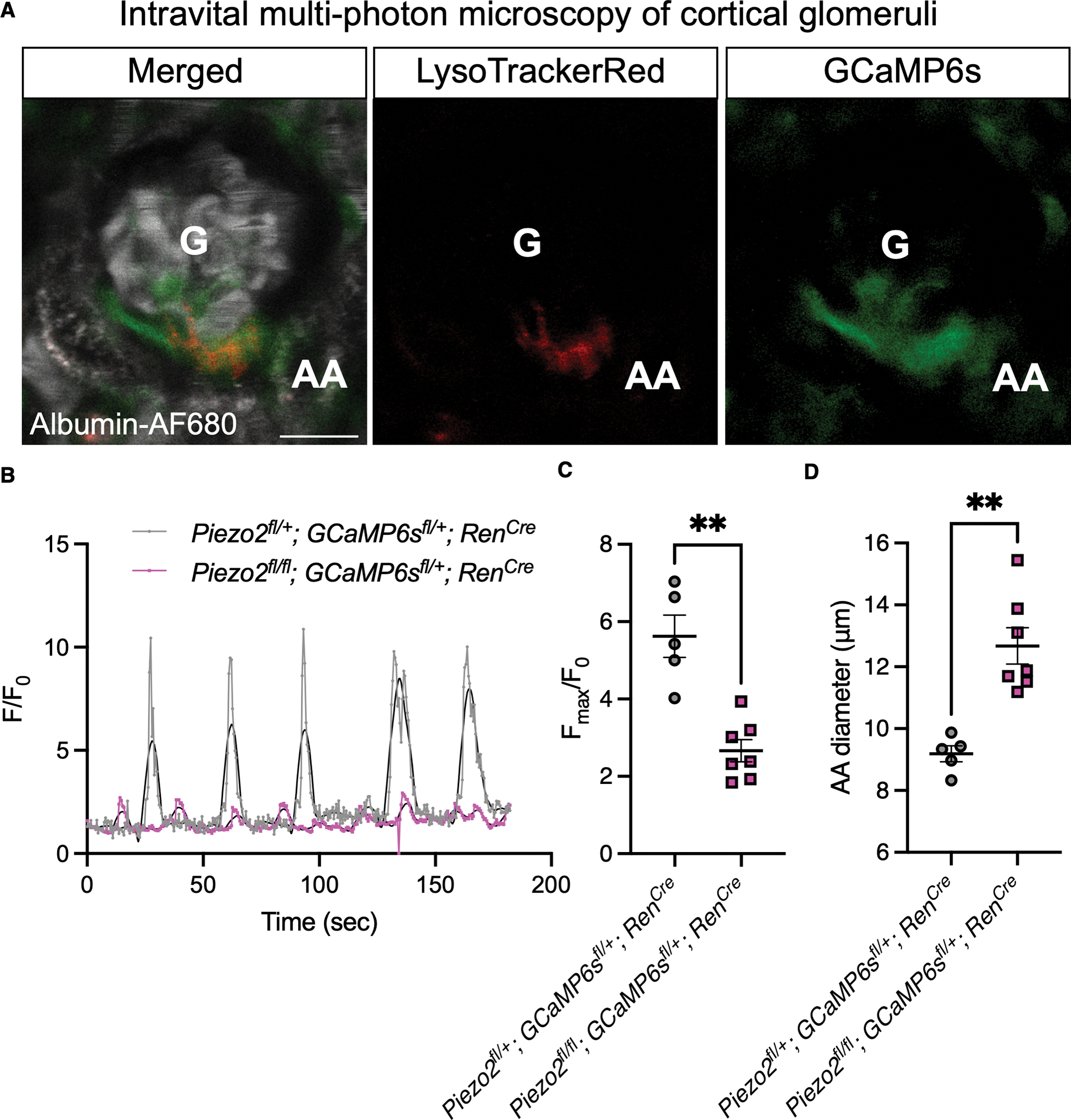
PIEZO2 regulates JG cell calcium oscillations *in vivo*. **A.** Representative images of glomerular (G) morphology including afferent arteriole (AA) vascular diameter (left), renin content (LysoTrackerRed, center), and calcium signaling (GCaMP6s, right) in living *Piezo2*^*fl/*+^*; GCamp6s*^*fl/*+^*; Ren*^*Cre*^ mouse kidney. Plasma was labeled with Alexa Fluor 680 (AF680)-conjugated albumin and injected i.v., scale = 50 μm. **B.** Intracellular calcium oscillations detected from GCaMP6s fluorescence normalized to baseline (F/F_0_) in single renin^+^ cells identified by LysoTracker Red labeling) in control (*Piezo2*^*fl/*+^*; GCamp6s*^*fl/*+^*; Ren*^*Cre*^, gray) versus PIEZO2 conditional knockout (*Piezo2*^*fl/fl*^*; GCamp6s*^*fl/*+^*; Ren*^*Cre*^, magenta) animals. Traces are representative of 5–10 cells each from N = 5 control and 7 conditional knockout animals. Smoothened traces (see STAR Methods) are overlaid in black. **C.** Maximal calcium signal normalized to baseline (F_max_/F_0_) in renin^+^ cells (Mann–Whitney: **p = 0.0025, U = 0; n = 5–10 cells each from N = 5 control and 7 conditional knockout animals). **D.** AA diameters (Mann–Whitney: **p = 0.0025, U = 1; n = 2–5 AA each from N = 5 control and 7 conditional knockout animals. Error bars represent mean ± s.e.m. See also Figure S8.

Intracellular calcium levels negatively regulate renin synthesis and release from JG cells through inversely calcium sensitive adenylyl cyclase.^16^ JG cells display intracellular calcium oscillations *in vivo* in response to tubuloglomerular feedback (TGF) and changes in myogenic tone in the adjacent afferent arteriole (vasomotion) that modulate renin at the microscale level.^21^ It has been hypothesized but not yet shown that JG cells directly sense vasomotion through MA ion channels. To observe intracellular calcium fluctuations in JG cells of living kidney cortex with an intact and functioning vascular network, we turned to multi-photon microscopy of live, anesthetized mice (Figure 4A).^58–60^ We measured the calcium dynamics of JG cells from *Piezo2*^*fl/fl*^*; GCaMP6s*^*fl/*+^*; Ren*^*Cre*^ mice and compared them to recordings from *Piezo2*^*fl/*+^*; GCaMP6s*^*fl/*+^*; Ren*^*Cre*^ heterozygous controls. JG cells at the terminal afferent arteriole of individual glomeruli were identified by GCaMP6s signal and uptake of LysoTracker Red dye, which labels JG cells based on the presence renin-containing secretory vesicle machinery,^60^ and were recorded for three minutes. Control animals displayed oscillations consistent with afferent arterioles undergoing constriction and dilation as previously reported (Figure 4B),^21,59,61^ validating the use of heterozygous animals as controls. Strikingly, we observed a massive reduction in the magnitude of intracellular calcium oscillations in conditional knockout animals (Figures 4B–C). Considering the well-established link between intracellular calcium in JG cells and control of renin transcription and secretion,^15^ our results are consistent with the mechanosensor PIEZO2 driving these functions in response to normal physiological mechanical forces generated by the renal vasculature.

The diameter of the glomerular afferent arteriole, the major strain vessel in the renal vasculature, was measured during *in vivo* imaging as a readout of local glomerular vascular function and vascular tone (Figure 4D). Conditional knockout animals featured consistently dilated afferent arterioles compared to controls, likely triggered by impaired calcium-dependent feedback mechanisms in the JG cells. The observed increase in diameter of the afferent arteriole suggests that PIEZO2 in renin-expressing cells might regulate glomerular hemodynamics and GFR, either directly or indirectly via signal propagation to adjacent contractile cells.

As a secondary assessment of PIEZO2 channel activity, we measured mechanically activated (MA) currents in response to controlled cellular indentation during whole-cell electrophysiology^25^ in primary cultured control and conditional knockout cells of renin lineage (Figures S8A–B). Remarkably, nearly all labeled cells displayed robust MA currents (Figure S8C). We were surprised that loss of PIEZO2, a rapidly inactivating MA ion channel, only affected the inactivation kinetics of the currents and remaining proportion of non-inactivating current (Figures S8D–G), suggesting loss of a rapidly inactivating component (*i.e.* an ion channel) without a loss of overall mechanosensitivity. Unlike our characterization of cells of renin lineage *in vivo*, we observed expression of *Piezo1* (Figure S8H), a phenomenon reported in primary cultures from the kidneys and other tissues.^18,62^ Consistent with this, loss of PIEZO1 and PIEZO2 largely ablated MA currents in the cultured cells (Figure S8I–J). These results provide a potential explanation for a previous study implicating PIEZO1 in renin release from JG cells that relied on cultured cells.^18^

Collectively, these data implicate mechanotransduction as a driving force of JG cell calcium oscillations *in vivo* and demonstrate that PIEZO2 is an essential sensor of vascular forces experienced by JG cells, even under naïve physiological conditions. Our findings in primary cultures establish that cells of renin lineage are broadly mechanosensitive and express functional PIEZO2, although this system is less useful for exploring PIEZO2 function in the absence of confounding MA channels such as PIEZO1. These results link together JG cell calcium dynamics with PIEZO2-dependent mechanotransduction.

### PIEZO2-dependent renin regulation is linked to modulation of GFR *in vivo*

**Figure 5.**
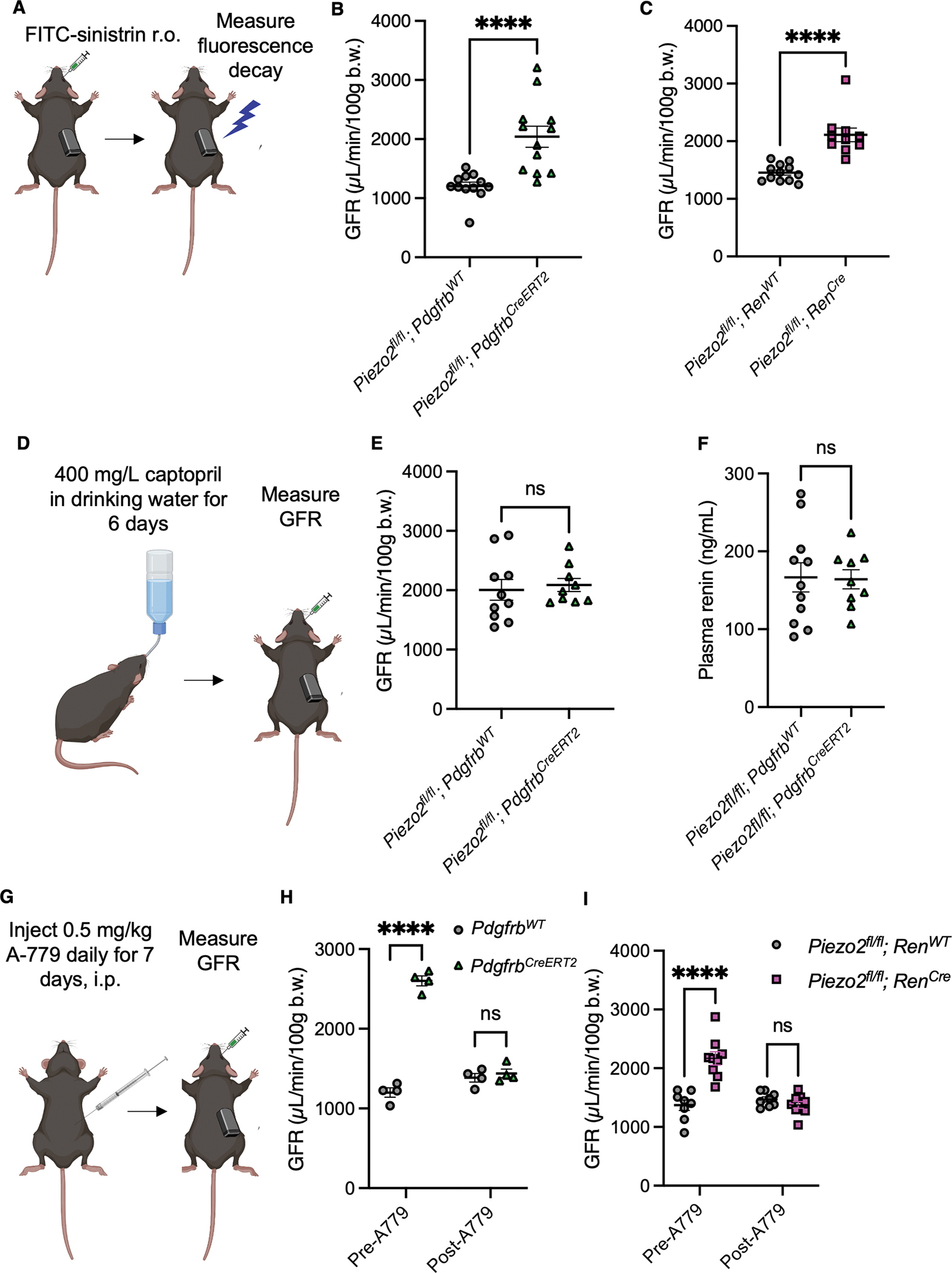
PIEZO2 regulates the GFR. **A.** GFR measurement in mice (created with BioRender.com). **B.** GFR (Mann–Whitney: ****p < 0.0001, U = 7; n = 12 *Pdgfrb*^*WT*^ and 12 *Pdgfrb*^*CreERT2*^ mice). **C.** GFR (Mann–Whitney: ****p < 0.0001, U = 1; n = 12 *Ren*^*WT*^ and 10 *Ren*^*Cre*^ mice. **D.** Captopril experiment (created with BioRender.com). **E.** GFR after captopril (Mann– Whitney: *p* = 0.4470, U = 35; n = 10 *Pdgfrb*^*WT*^ and 9 *Pdgfrb*^*CreERT2*^ mice). **F.** Plasma renin levels after captopril (Mann–Whitney: *p* = 0.8820, U = 47; n = 11 *Pdgfrb*^*WT*^ and 9 *Pdgfrb*^*CreERT2*^ mice). **G.** A779 MAS blockade experiment (created with BioRender.com). **H.** GFR before (pre-A779) and after (post-A779) treatment with A779 (two-way ANOVA: ****p_*interaction*_ < 0.0001, F(1,6) = 248.9; Sidak’s multiple comparisons: ****p_*pre-A779*_ < 0.0001, *p*_*post-A779*_ = 0.7724; n = 4 *Pdgfrb*^*WT*^ and 4 *Pdgfrb*^*CreERT2*^ mice). **I.** GFR before (pre-A779) and after (post-A779) treatment with A779 (two-way ANOVA: ****p_*interaction*_ < 0.0001, F(1,15) = 28.69; Sidak’s multiple comparisons: ****p_*pre-A779*_ < 0.0001, *p*_*post-A779*_ = 0.6662; n = 8 *Ren*^*WT*^ and 9 *Ren*^*Cre*^ mice). Each experiment was performed on at least two cohorts of mice, and error bars represent mean ± s.e.m. See also Figure S9.

An important secondary function of renin and the RAAS is to modulate the GFR in response to TGF and renal hemodynamics through mechanisms acting on the glomerular vasculature.^63,64^ We measured GFR through intravenous administration of FITC-sinistrin followed by continuous measurement of fluorescence signal decay.^66^ *Piezo2*^*fl/fl*^*; Pdgfrb*^*CreERT2*^ mice exhibited glomerular hyperfiltration (Figures 5A–B). The GFR values of the conditional knockout mice were very high and similar to those observed in genetic models of hyperfiltration.^65^ Driven by the observation of dilated afferent arterioles with loss of PIEZO2, we sought a mechanistic explanation. Initially, we examined the possibility that our mice had developed kidney disease. We found that the GFR of the *Piezo2*^*fl/fl*^*; Pdgfrb*^*CreERT2*^ mice declined as expected with age^66,67^ (Figure S9A–B),suggesting that the observed phenotype was not due early stages of kidney disease. Additionally, the conditional knockout mice did not exhibit elevated urinary albumin or blood urea nitrogen (BUN, Figures S9C–D). Under healthy conditions, mesangial cells are also thought to regulate the GFR through their contractility.^54,65^ Counter to this idea, we observed hyperfiltration in *Piezo2*^*fl/fl*^*; Ren*^*Cre*^ conditional knockouts (Figure 5C). Importantly, loss of PIEZO1 and PIEZO2 in peripheral neuronal baroreceptors using *Piezo1*^*fl/fl*^*; Piezo2*^*fl/fl*^*; SNS*^*Cre*^ did not induce a GFR phenotype (Figure S9E). We also considered whether anatomical defects in the kidneys could account for the hyperfiltration. Examination of age-matched PAS- and H&E-stained sections showed that neither *Piezo2*^*fl/fl*^*; Pdgfrb*^*CreERT2*^ nor *Piezo2*^*fl/fl*^*; Ren*^*Cre*^ kidneys exhibited distinct histological features compared to controls (Figures S9F–I).

We next postulated that the elevated GFR could be related to the afferent arteriolar dilation we observed in our imaging experiments. Of note, infusions of the angiotensin peptide Ang(1–7) have been shown to increase the GFR via MAS Receptor signaling, opposing some of the effects of Ang II signaling via AT receptors.^68–71^ The Ang(1–7)/MAS axis is also reported to regulate the GFR under salt-depleted conditions,^72,73^ which tracks with the elevated renin required to increased Ang(1–7) production that drives relaxation of the afferent arteriole.^74^ Ang(1–7) can be produced from Ang I, II, or (1–9) by several specialized peptidases, such as angiotensin converting enzyme 2 (ACE2), which is the most potent enzyme to form Ang(1–7) from Ang II.^68,75,76^ An increase in Ang(1–7) can also occur after treatment with ACE inhibitors such as captopril that inhibit its degradation.^77,78^ MAS receptor knockout mice develop hyperfiltration, which conflicts with the reported effects of Ang(1–7) infusions on GFR that are MAS receptor-dependent; however, the hyperfiltration coincides with fibrotic changes in the kidney that are thought to drive the increase in GFR.^68^ This makes the role of MAS signaling difficult to study using a genetic model of MAS Receptor deletion.

We examined whether the elevated GFR could be mimicked through elevation of renin in the absence of elevated Ang II using captopril. Like other ACE inhibitors, captopril can increase the GFR in normotensive animal models.^77–82^ This effect is reported to be downstream of elevated Ang(1–7).^82^ We observed that captopril administration (Figure 5D) elevated the GFR of wild-type mice such that it was not significantly different from the *Piezo2*^*fl/fl*^*; Pdgfrb*^*CreERT2*^ littermates (Figure 5E). Conditional knockout animals did not develop further increases in GFR beyond what we initially observed (Figure 5E). As expected, captopril increased plasma renin (Figure 5F) and suppressed aldosterone in mice of both genotypes (Figure S9J). The renin levels observed in *Piezo2*^*fl/fl*^*; Pdgfrb*^*CreERT2*^ animals treated with captopril (Figure 5F) were higher than those observed in untreated animals (Figure 3A), despite GFR values within a similar range (Figure 5B). Thus, increased renin drives glomerular hyperfiltration in mice with intact PIEZO2, suggesting a “ceiling effect” of renin on GFR: the higher renin levels elicited by captopril do not raise the GFR further than that observed in the untreated conditional knockout mice.

We tested whether enhanced MAS signaling via Ang(1–7) contributed to elevated GFR in the conditional knockout mice. We measured GFR before and after blocking MAS signaling via the pharmacological antagonist A779 (Figure 5G). Strikingly, A779 rescued GFR of *Piezo2*^*fl/fl*^*; Pdgfrb*^*CreERT2*^ and *Piezo2*^*fl/fl*^*; Ren*^*Cre*^ mice to normal levels and had no effect on the GFR of controls (Figures 5H–I), supporting that MAS signaling underlies the elevation in GFR caused by loss of PIEZO2. In light of the previously reported fibrotic kidney changes in MAS Receptor knockout mice and reported crosstalk of Ang(1–7) with AT2R receptors,^68,79^ future studies using inducible and cell-type specific knockout of MAS will help to differentiate roles of Ang(1–7) and MAS signaling on GFR *in vivo*.

Angiotensin-related peptides are notoriously difficult to measure owing to their short lifetimes in tissue and circulation. To investigate how loss of PIEZO2 might elevate Ang(1–7) to drive MAS signaling, we measured the activities of a variety of angiotensin peptidases, as activity levels of the circulating peptidases are reported to track with levels of the peptides.^75,83–85^ We observed a significant elevation in ACE2 activity in conditional knockout mice (Figure S9K). This suggests that the increased GFR may be due to Ang(1–7) formed from increased conversion of Ang II (in turn produced by increased renin and RAS activation) by ACE2. By contrast, we did not observe altered prolyl-endopeptidase (PEP), aminopeptidase A (APA), or ACE activity, which would affect circulating Ang II (Figure S9L). Our findings support a molecular pathway by which PIEZO2-dependent renin regulation modulates the GFR via ACE2/Ang(1–7)/MAS signaling and provide a potential explanation for why Ang II is not elevated in our models under naïve conditions.

### PIEZO2 governs RAAS in acute and chronic volume challenges

A primary function of the RAAS is to conserve bodily salt and water when blood volume is depleted (hypovolemia), which can occur acutely during blood loss or chronically when dietary sodium and/or water is withheld. Renin and the RAAS components are suppressed when blood volume is normal (normovolemia) or high (hypervolemia), which can occur with excessive intake or impaired excretion of fluids and sodium. We reasoned that PIEZO2 would participate in the responses to acute and chronic changes in blood volume that regulate renin and the RAAS.

**Figure 6.**
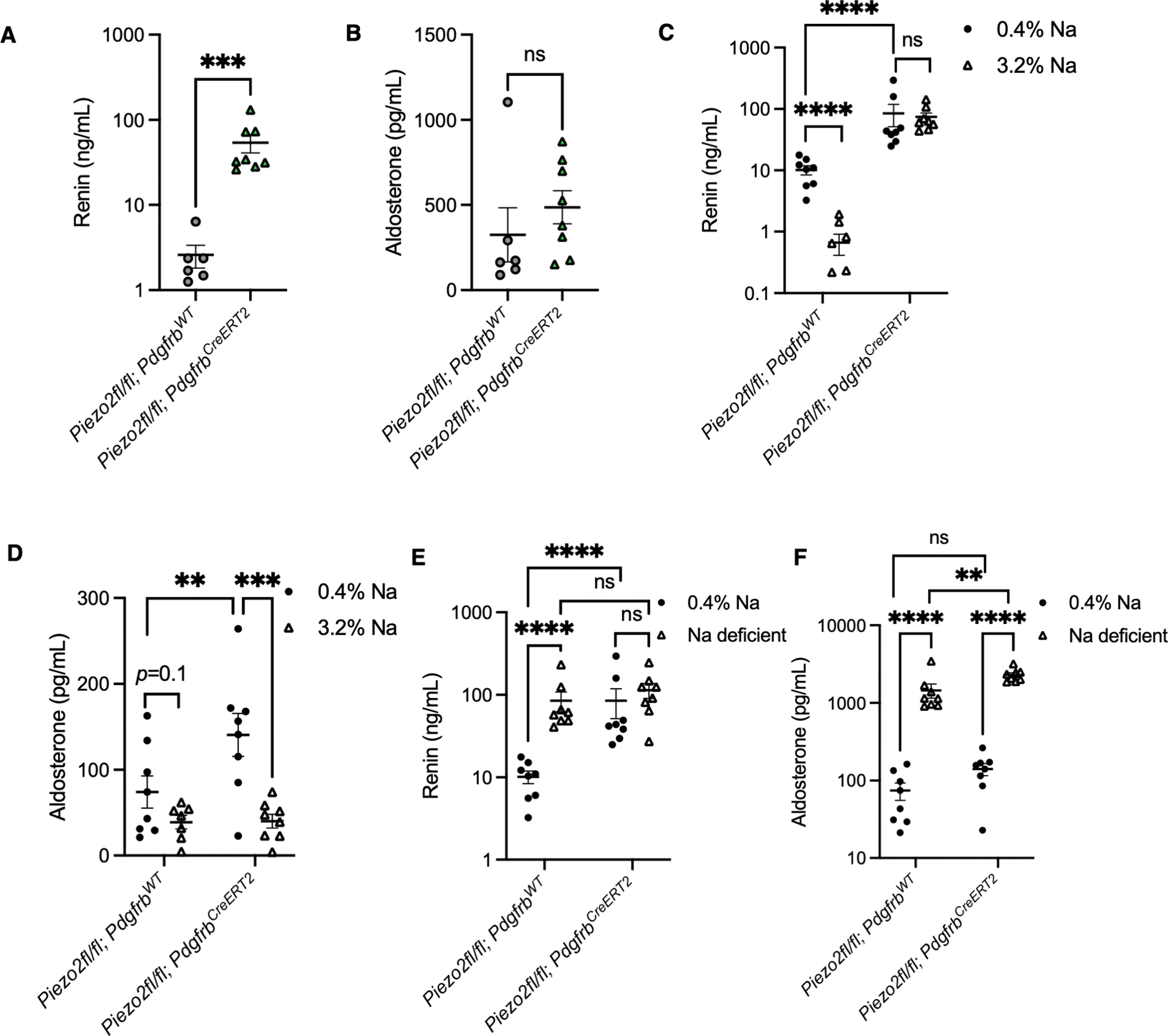
PIEZO2 contributes to acute hypervolemia and dietary sodium-induced changes in renin levels. **A.** Plasma renin levels after saline (Mann–Whitney: ***p = 0.0007, U = 0; n = 6 *Pdgfrb*^*WT*^ and 8 *Pdgfrb*^*CreERT2*^ mice). **B.** Plasma aldosterone levels after saline (Mann–Whitney: *p* = 0.1419, U = 12; n = 6 *Pdgfrb*^*WT*^ and 8 *Pdgfrb*^*CreERT2*^ mice). **C.** Plasma renin levels (two-way ANOVA: ****p_*interaction*_ < 0.0001, F(1,26) = 29.45434; Uncorrected Fisher’s LSD (left to right): ****p < 0.0001, ****p < 0.0001, *p* = 0.6164; n = 8 *Pdgfrb*^*WT*^ control, 6 *Pdgfrb*^*WT*^ high sodium, 8 *Pdgfrb*^*CreERT2*^ control, and 8 *Pdgfrb*^*CreERT2*^ high sodium mice). **D.** Plasma aldosterone levels in mice from **C** (two-way ANOVA: ***p_*genotype*_ = 0.0005, F(1,26) = 15.83434; Uncorrected Fisher’s LSD (left to right): *p* = 0.1, **p = 0.0092, ***p = 0.0002; n = 8 *Pdgfrb*^*WT*^ control, 6 *Pdgfrb*^*WT*^ high sodium, 8 *Pdgfrb*^*CreERT2*^ control, and 8 *Pdgfrb*^*CreERT2*^ high sodium mice). **E.** Plasma renin levels (two-way ANOVA: **p_*interaction*_ = 0.0030, F(1,28) = 10.55225; Uncorrected Fisher’s LSD (left to right): ****p < 0.0001, ****p < 0.0001, *p* = 0.8084, *p* = 0.4351; n = 8 *Pdgfrb*^*WT*^ control, 8 *Pdgfrb*^*WT*^ sodium deficient, 8 *Pdgfrb*^*CreERT2*^ control, and 8 *Pdgfrb*^*CreERT2*^ sodium deficient mice). **F.** Plasma aldosterone from mice in **E** (two-way ANOVA: *p_*interaction*_ = 0.0305, F(1,28) = 5.194771; Uncorrected Fisher’s LSD (left to right): ****p < 0.0001, *p* = 0.7840, **p = 0.0016, ****p < 0.0001; n = 8 *Pdgfrb*^*WT*^ control, 8 *Pdgfrb*^*WT*^ sodium deficient, 8 *Pdgfrb*^*CreERT2*^ control, and 8 *Pdgfrb*^*CreERT2*^ sodium deficient mice). Each experiment was performed on at least two independent cohorts of mice, and error bars represent mean ± s.e.m. Data from **C** and **E** were subjected to a log-transform prior to statistical analysis. Data from control mice are replotted in **C-D** vs. **E-F** with statistics run separately.

To this end, we measured RAAS components in *Piezo2*^*fl/fl*^*; Pdgfrb*^*CreERT2*^ and littermate controls subjected to four distinct blood volume challenges: acute hypervolemia, chronic hypervolemia, chronic hypovolemia, and acute hypovolemia. We first modeled acute hypervolemia using intraperitoneal injection of saline.^86^ Plasma renin levels in control mice were close to the assay’s limit of detection (Figure 6A) and lower than in previous unchallenged experiments (Figure 3A). A lack of renin suppression was observed in conditional knockout mice compared to controls (Figure 6A). We did not observe any significant difference in aldosterone levels (Figure 6B), consistent with a lack of aldosterone stimulation under naïve (Figure 3B) or hypervolemic conditions.

Subsequently, we measured RAAS components in control and conditional knockout mice after chronically manipulating blood volume with either excess (hypervolemia) or near-absence (hypovolemia) of dietary sodium.^.79^ With chronic hypervolemia, we observed a suppression of renin in control mice, whereas the renin levels of conditional knockouts displayed an insensitivity to the challenge (Figure 6C). Aldosterone levels trended toward suppression in control mice and were significantly lower in the conditional knockouts (Figure 6D). With chronic hypovolemia, we observed elevated renin and aldosterone in controls as expected (Figures 6E–F). In the conditional knockout mice, renin levels were heightened and unchanged between dietary conditions, demonstrating that PIEZO2 is essential for responsiveness of renin levels in chronic volume challenge. Intriguingly, aldosterone levels were still robustly induced in conditional knockout mice (although higher than control mice in the sodium-deficient condition), suggesting that other mechanisms regulate aldosterone in this challenge.

**Figure 7.**
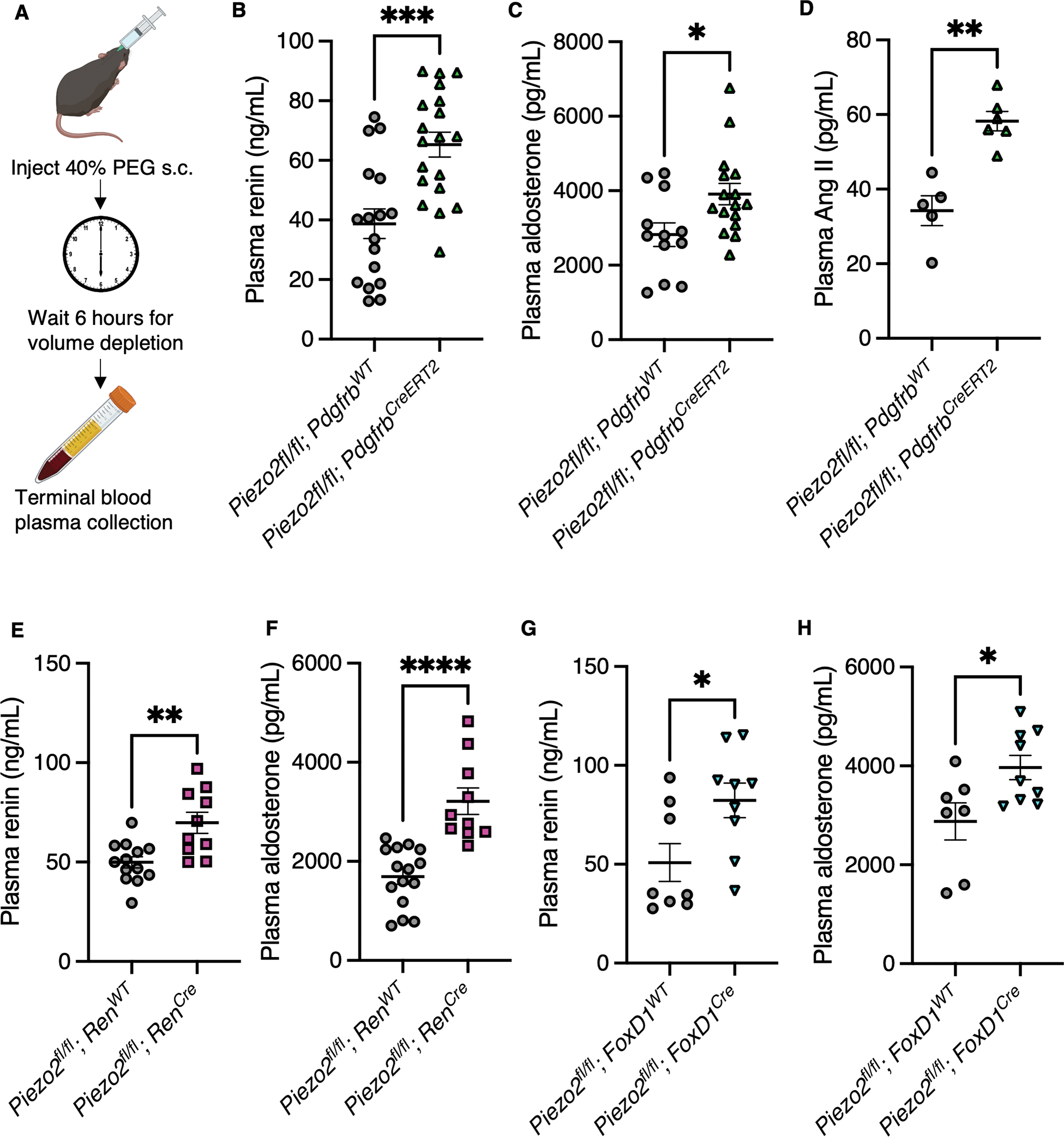
PIEZO2 modulates the hormonal response to acute hypovolemia. **A.** PEG model of hypovolemia (created with BioRender.com). **B.** Plasma renin levels (Mann–Whitney: ***p = 0.0003, U = 53; n = 17 *Pdgfrb*^*WT*^ and 19 *Pdgfrb*^*CreERT2*^ mice). **C.** Plasma aldosterone levels (Mann–Whitney: *p = 0.0257, U = 48; n = 12 *Pdgfrb*^*WT*^ and 16 *Pdgfrb*^*CreERT2*^ mice). **D.** Plasma Ang II levels (Mann–Whitney: **p = 0.0043, U = 0; n = 5 *Pdgfrb*^*WT*^ and 6 *Pdgfrb*^*CreERT2*^ mice). **E.** Plasma renin levels (Mann–Whitney: **p = 0.0025, U =18; n = 13 *Ren*^*WT*^ and 10 *Ren*^*Cre*^ mice). **F.** Plasma aldosterone levels (Mann–Whitney: ****p < 0.0001, U =2; n = 15 *Ren*^*WT*^ and 10 *Ren*^*Cre*^ mice). **G.** Plasma renin levels (Mann–Whitney: *p = 0.0360, U =14; n = 8 *FoxD1*^*WT*^ and 9 *FoxD1*^*Cre*^ mice). **H.** Plasma aldosterone levels (Mann–Whitney: *p = 0.0418, U =12; n = 7 *FoxD1*^*WT*^ and 9 *FoxD1*^*Cre*^ mice). Each experiment was performed on at least two independent cohorts of mice, and error bars represent mean ± s.e.m. See also Figures S10 and S11.

To induce acute hypovolemia, we subjected *Piezo2*^*fl/fl*^*; Pdgfrb*^*CreERT2*^ and littermate controls to the polyethylene glycol (PEG)-evoked hypovolemia model^89^ (Figure 7A), where subcutaneous PEG causes a volume depletion without directly affecting salt balance,^87^ as with the loop diuretic furosemide.^88^ PEG-injected *Piezo2*^*fl/fl*^*; Pdgfrb*^*CreERT2*^ mice exhibited an exaggerated hormonal response to hypovolemia, with elevated renin, Ang II, and aldosterone compared to littermate controls (Figures 7B–D). The renin levels observed here were greater than that under normovolemia and potentially explain the stronger induction of Ang II and aldosterone in conditional knockouts that were correlated with renin (Figures S10A–B). Furthermore, these findings were reminiscent of our observations in the chronic hypovolemia model (Figures 6E–F), with the main difference being that acute hypovolemia triggered induction of renin in the conditional knockouts. The acute model differs from the chronic model in the rapidity of onset and severity of the challenge, which might engage other renin-stimulatory pathways acting in parallel with PIEZO2. The results were phenocopied with the *Piezo2*^*fl/fl*^*; Ren*^*Cre*^ and *FoxD1*^*Cre*^ strains (Figures 7E–H), suggesting that loss in the renin lineage alone was sufficient to drive the phenotypic effect. Like our earlier findings under naïve conditions, loss of both PIEZOs using *Piezo1*^*fl/fl*^*; Piezo2*^*fl/fl*^*; Pdgfrb*^*CreERT2*^ had no further effect beyond loss of PIEZO2 (Figure S10C). No difference was observed between conditional knockout and controls with dual loss of PIEZO1 and PIEZO2 in peripheral neuronal baroreceptors (Figure S10D). We conclude from these four different experiments to manipulate blood volume that PIEZO2 acts as a key suppressor of renin in hyper- and normovolemia and serves as a brake on the hormonal response to hypovolemia.

### PIEZO2 contributes to renal baroreceptor functions independently of intact sympathetic efferent and macula densa signaling

We next sought to determine how the contribution of PIEZO2 to the induction of the RAAS is weighted against that of the other pathways regulating renin during hypovolemia. To address this question, we designed an experiment in which we ablated sympathetic nerves using the drug 6-hydroxydopamine (6-OHDA)^89,90^ and acutely blocked prostaglandin synthesis using the cyclooxygenase-1 and -2 inhibitor indomethacin.^12,13,89^ After chemical sympathectomy and indomethacin injection, *Piezo2*^*fl/fl*^*; Ren*^*Cre*^ mice and controls were subjected to PEG or saline treatment (Figure S11A). We observed a near-complete loss of TH^+^ sympathetic nerve fibers in the kidney, indicating the 6-OHDA treatment was highly effective (Figures S11B–C). In the control mice, we found that plasma renin levels were strongly suppressed with blockade of both sympathetic and macula densa prostaglandin signaling (Figure S11D), suggestive of a successful blockade of renin stimulatory pathways. Plasma renin in the PEG-treated wild-type mice was still sensitive to PEG hypovolemia albeit to a lesser extent than when compared to mice with intact sympathetic and prostaglandin signaling (Figure S11D). In normovolemic *Piezo2*^*fl/fl*^*; Ren*^*Cre*^ mice, renin levels were largely unaffected by the blockade and akin to that of wild-type, normovolemic mice with intact sympathetic and macula densa signaling (refer to Figures 3A and 3H). In PEG-injected conditional knockout mice, renin levels were elevated beyond that of littermate controls and comparable to hypovolemic *Piezo2*^*fl/fl*^*; Ren*^*Cre*^ mice with intact sympathetic and macula densa signaling, suggesting that loss of mechanotransduction in cells of renin lineage dysregulates the RAAS independently of the sympathetic and macula densa prostaglandin pathways (Figure S11D). We were surprised to observe that renin levels were still sensitive to acute hypovolemia. Aldosterone levels were similarly exacerbated by hypovolemia (Figure S11E). We speculate that a fourth (i.e., PIEZO2-independent) pathway may stimulate the RAAS, such as a reduction in adenosine/ATP signaling,^14^ or that the pharmacological blockade was partial. These findings demonstrate that PIEZO2 acts as a brake on renin induction during hypovolemia, even when sympathetic efferent and macula densa signaling are suppressed.

## Discussion

Here we present a cellular and molecular mechanism for how mechanotransduction regulates JG cell function and RAAS, uncovering an essential role for PIEZO2 in the regulation of renin under naïve conditions and during volume expansion and depletion. Our work expands on the roles of PIEZO proteins to highlight them as general effectors of baroreceptor function throughout the body.

The discovery of elevated GFR with loss of PIEZO2 in cells of renin lineage was surprising. We propose that the observed PIEZO2-dependent increase in ACE2 activity drives hyperfiltration through increased Ang(1–7)/MAS receptor signaling. Our evidence for this supposition is two-fold. Directly, we demonstrate that MAS Receptor blockade restores normal GFR of conditional knockouts without affecting control animals. Indirectly, our *in vivo* imaging experiments showed constitutively larger afferent arteriolar diameters of conditional knockouts compared to control mice. Ang(1–7)/MAS signaling dilates the afferent arterioles to drive an elevation in GFR,^69,71,90^ although it remains to be seen whether circulating and/or local Ang(1–7) are elevated in our PIEZO2 deficient models. Measurement of renal and circulating Ang(1–7), although highly technically challenging due to the need to distinguish between different angiotensin peptides with extraordinarily short lifetimes in the circulation, would directly answer this question. Moreover, our study raises the question of how loss of PIEZO2 drives elevated ACE2 activity. The regulation of ACE2 is poorly understood and represents a burgeoning field.^93^ For example, elevated ACE2 activity could represent a compensatory response to prevent excess Ang II when renin is elevated.

It is challenging to reconcile the minute changes in glomerular hemodynamics, classically observed using nephron micropuncture of larger animals that first established the role of renin and JG cells in TGF and GFR,^93^ with whole-organism effects on the RAAS established through genetic models in mice. Our work links regulation of renin to *in vivo* JG cell calcium dynamics through PIEZO2, providing a molecular explanation for how these cells sense and integrate microscale changes to drive major shifts in physiology.

On another note, our work could lead to a better understanding of how JG cell mechanotransduction may affect the pathophysiology of poorly understood kidney diseases such as chronic kidney disease of unknown etiology (CKDu), which is primarily observed in agricultural workers who are repeatedly subjected to a combination of heat stress and volume depletion.^94^

### Limitations of the study:

An important caveat of our study is our inability to deplete PIEZO2 from JG cells while sparing efferent arteriolar cells of renin lineage using existing approaches, given that the *Ren*^*Cre*^ line can potentially target both cell types. A recent study using a different Cre driver line based upon the *Ren1d* gene did not observe a role for PIEZO2 in the regulation of local renin.^95^ Using identical methods to their study, we observed an increase in local renin in our conditional knockouts that use the *Ren1c* gene to drive Cre, suggesting that even minor differences between Cre lines and/or strains, which could affect Cre-dependent conditional gene knockout amongst renal cell types, could play important roles in the phenotypes we observe and present a possibility we cannot completely rule out. Our *in vivo* imaging, where JG cells were visually identified through anatomical location and dye uptake, is highly suggestive of a primary role for PIEZO2 in JG cells but cannot completely exclude these other cells. Development of genetic tools to manipulate each cell type separately with ample coverage for loss-of-function studies will be useful to investigate mechanisms of renin regulation and GFR control and enable measurement of the myogenic response of PIEZO2^+^ efferent arteriolar mural cells. Of note, the role of PIEZO2 in contractile mesangial cells, which we found to be dispensable for regulation of renin, remains to be understood. We speculate that mesangial PIEZO2 (or induction of PIEZO1 expression under disease conditions) could play important roles in conditions affecting the mechanical microenvironment of the glomerulus (*e.g.,* diabetic nephropathy with glomerulosclerosis) or in functions associated with mesangial cell contractility.^29,30^

Another question raised by our work is the link between renal PIEZO2 and blood pressure. Our study focuses mainly on renin; however, the observed elevation in blood pressure was surprising given the lack of elevated circulating Ang II. One possible explanation lies within the measurement itself. While the VPR method of blood pressure is comparable to telemetry for many purposes, it provides only a single-point snapshot of blood pressure, which varies even on the scale of a few cardiac cycles.^51,52^ Long-term telemetry recordings will be necessary to fully investigate a link between renal PIEZO2 and blood pressure, especially in mice that are still within the normotensive range such as ours. And as with Ang(1–7), the ability to accurately quantify Ang II over several time points would address whether subtle or transient elevations in Ang II might drive the small non-hypertensive increase in blood pressure, even with competing elevations in Ang(1–7). While our experiments provide substantial mechanistic insight into how mechanosensation controls renin, questions remain for future studies to explore its relationship to angiotensin peptides and blood pressure.

## Resource Availability

### Lead contact:

Correspondence to Rose Z. Hill (hillros@ohsu.edu).

### Materials availability:

No new materials were generated by this study.

### Data and code availability:

- Single nucleus RNA-seq data including the Cell Ranger output files and Seurat object have been deposited to NCBI Gene Expression Omnibus (GEO) under accession number GSE280628 and is publicly available in NCBI GEO as of the date of publication. snRNA-seq data are publicly available to interactively browse^96^ at https://nelsonlab.shinyapps.io/Pdgfrb_INTACT/.
- All individual data points are presented as dot plots in the Figures or Supplementary Figures. All raw and supporting data reported in this paper will be shared by the lead contact upon request.
- All original code and scripts have been deposited at Zenodo at DOI: 10.5281/zenodo.17487554 and is publicly available as of the date of publication.
- Any additional information required to reanalyze the data reported in this paper is available from the lead contact upon request.

## STAR Methods

### EXPERIMENTAL MODEL AND STUDY PARTICIPANT DETAILS

All experiments were approved by the Scripps Research Animal Care and Use Committee (Animal Use Protocol 08–0136). Mice were kept in standard housing with a 12-h light–dark cycle set with lights on from 6 am to 6 pm, with the room temperature kept around 22 °C, and humidity between 30% and 80% (not controlled). Mice were kept on pelleted paper bedding and provided with paper square nestlets and polyvinyl chloride pipe enrichment with *ad libitum* access to food and water. Littermate mice were used for all experiments. For all experiments, male and female mice were used and pooled. Mouse ages ranged from 12 to 20 weeks for all experiments unless otherwise indicated in the figure legends or respective STAR Methods section. All mice received metal identification tags (National Band & Tag, 1005–1) on the right ear when they were between 18 and 30 days old, except for *in vivo* imaging experiments where mice were genotyped at 16–18 days of age via ear punch without tag. After weaning between 21 and 30 days of age, mice were co-housed in groups of 2–5 littermates of the same sex. Genotyping was performed in-house by PCR from tail or ear snip DNA samples using guidelines and primer sequences from Jackson Laboratory or was performed by Transnetyx. The following strains of mice were used and maintained in the laboratory on an inbred background: *Piezo1*^*tdTomato*^ (*B6;129-Piezo1*^*tm1.1Apat/*^*J*; Jackson Laboratories 029214) and *Piezo2*^*EGFP-IRES-Cre*^ (*B6(SJL)-Piezo2*^*tm1.1(cre)Apat*^*/J*; Jackson Laboratories 027719). The following strains of mice were maintained on a *Ren1* monogenic C57BL6/J background: *Piezo1*^*fl/fl*^ (*B6.Cg-Piezo1 *^*tm2.1Apat*^*/J*; Jackson Laboratories 029213), *Piezo2*^*fl/fl*^ (*B6(SJL)-Piezo2*^*tm2.2Apat*^*/J*, Jackson Laboratories 027720), *Ai9*^*fl/fl*^ (*B6.Cg-Gt(ROSA)26Sor*^*tm9(CAG-tdTomato)Hze*^*/J*; Jackson Laboratories 007909), *Ai14*^*fl/fl*^ (*B6.Cg-Gt(ROSA)26Sor*^*tm14(CAG-tdTomato)Hze*^*/J*; Jackson Laboratories 007914), *Pdgfrb*^*CreERT2*^ (*B6.Cg-Pdgfrb*^*tm1.1(cre/ERT2)Csln*^*/J*, Jackson Laboratories 030201), *FoxD1*^*Cre*^ (*B6;129S4-Foxd1*^*tm1(GFP/cre)Amc*^*/J*, Jackson Laboratories 012463), *GCaMP6s*^*fl/fl*^ (B6J.Cg-*Gt(ROSA)26Sor*^*tm96(CAG-GCaMP6s)Hze*^/MwarJ, Jackson Laboratories 028866); *Ren*^*Cre*^ and *Ren*^*CreER*^ (*Ren1c*^*Cre*^ and *Ren1c*^*CreER*^, gifts from Drs. Kenneth Gross and Stuart Shankland), and *SNS*^*Cre*^ (*Tg(Scn10a-cre)*^*1Rkun*^, a gift from Dr. Rohini Kuner, MGI: 3042874). Conditional knockout lines were maintained by crossing a homozygous floxed Cre-expressing mouse (homozygous for one or more indicated floxed alleles) with homozygous floxed mate. All strains are commercially available except for *SNS*^*Cre*^, *Ren*^*Cre*^, and *Ren*^*CreER*^. For experiments involving the *Pdgfrb*^*CreERT2*^ line, recombination was achieved with once-daily intraperitoneal injection of 100 mg per kg body weight tamoxifen (Sigma-Aldrich, T5648) dissolved in 0.22-μm sterile-filtered corn oil delivered to both Cre-expressing and control mice over five consecutive days. Mice were used four or more weeks after tamoxifen administration to ensure adequate time for Cre activity and protein turnover. *Pdgfrb*^*CreERT2*^ (*B6.Cg-Tg(Pdgfrb-cre/ERT2)6096Rha/J*, Jackson Laboratories 029684) mice were crossed to *CAG-Sun1-sfGFP* (B6;129-Gt(ROSA)26Sor^tm5(CAG-Sun1/sfGFP)Nat^/J, Jackson Laboratories 021039) mice to generate the *Pdgfrb*^*CreERT2*^-INTACT (Isolation of Nuclei TAgged in specific Cell Types) mice used for snRNA-Seq experiments.^37,38^ Two male *Pdgfrb*^*CreERT2*^-INTACT mice were i.p. injected with 1 mg tamoxifen daily for five days to induce the sfGFP expression.

### METHOD DETAILS

#### Single molecule fluorescent in situ hybridization (smFISH):

For mouse experiments, kidneys were removed immediately, embedded in optimal cutting temperature compound (OCT, Sakura), and flash-frozen in liquid nitrogen. For human kidney biopsies, 5 μm-thickness formalin-fixed paraffin embedded (FFPE) kidney sections were obtained from the Kidney Translational Resource Center at Washington University and processed using the manufacturer’s instructions for FFPE slides. Tissue from a single anonymous White/Caucasian male donor aged 46 was used. Informed consent and IRB approval for human kidney samples was obtained by the KTRC. The protocol for RNAscope Multiplex Fluorescent Reagent Kit V2 (ACDBio, 323100) was followed exactly according to the instructions for fresh-frozen and FFPE tissue. Protease IV was applied for 30 min for mouse tissue. For human kidney sections, manufacturer’s instructions were followed exactly for FFPE kidney tissue. Probes (all from ACDBio) for mouse *Piezo1* (#400181), mouse *Piezo2* (#400191), mouse *Piezo2*-E43-E45 (#439971), mouse *Ren1* (#433461), mouse *Pdgfrb* (#411381), mouse *Pecam1* (#316721), human *PIEZO1* (#485101), human *PIEZO2* (#449951), and human *PDGFRB* (#548991) were applied to detect transcript. The manufacturer’s 3-plex negative control probe (#320871) was used in each experiment to detect non-specific signal. Displayed images were uniformly cropped from the original images on which quantification was performed.

#### Immunohistochemistry (IHC):

For *Piezo1*^*tdTomato*^ and *Piezo2*^*GFP-Cre*^ IHC experiments, tissues were processed using a modified protocol to preserve signal.^97^ In brief, fresh-frozen kidneys were embedded in OCT and sectioned at 20 μm. Sections were post-fixed on slides in cold 4% paraformaldehyde (PFA) in PBS for 10 min at room temperature and quenched using 20 mM glycine and 75 mM ammonium chloride with 0.1% v/v Triton X-100 in PBS (PBST) for 10 min. Slides were washed in PBS and then incubated in blocking buffer (0.6% w/v fish skin gelatin with 0.05% w/v saponin in PBS with 5% v/v normal goat or donkey serum) for 1 h at room temperature. Slides were incubated in primary antibodies overnight at 4 °C in blocking buffer without serum: AlexaFluor 647-conjugated FluoTag-X4 anti-RFP single domain antibody (Nanotag, N0404, 1:100) or chicken anti-GFP (Aves Labs, GFP1010, 1:1000). When conjugated nanobody was used, slides were washed in PBS and mounted in SlowFade Diamond immediately prior to imaging. For GFP staining experiments, slides were washed in PBS, and then incubated in goat anti-chicken Alexa Fluor 488 secondary antibody (Life Technologies, A11039, 1:1000) in blocking buffer 1 h at room temperature. Samples were washed in PBS, counterstained with 1:30,000 TO-PRO-3 Iodide (Life Technologies, T3605), and then mounted in SlowFade Diamond mounting medium (Life Technologies, S36967) and sealed with nail polish prior to imaging.

For conventional IHC of non-conjugated tdTomato, marker proteins, and/or renin, fresh-frozen kidneys were embedded in OCT and sectioned at 20 μm. Sections were post-fixed on slides for 15 minutes at 4 °C in 4% v/v PFA-PBS, briefly rinsed in PBS, washed for 10 min in 0.3% v/v Triton X-100 in PBS (PBST), then blocked for 1 h in 5% v/v normal goat serum in 0.3% PBST. Sections were incubated overnight at 4 °C in rabbit anti-renin (Abcam, ab212197, 1:250), rat anti-PECAM1 (Sigma Aldrich, CBL-1337–1, 1:1000), rabbit anti-NPHS2 (Abcam, ab50339, 1:1000), or rabbit anti-RFP (Rockland, 600–401-379, 1:1000) in 0.3% PBST with 1% NGS. Sections were washed in PBS and incubated in 1:1,000 goat anti-rabbit AlexaFluor 647 (Life Technologies, A21245) and/or goat anti-rat 488 (Life Technologies, A11006) for 1 h at room temperature. Tissues were rinsed in PBS, mounted in HIGHDEF IHC Fluoromount (Enzo), and sealed with nail polish. For both smFISH and IHC, all samples were imaged on either a Nikon A1 or AX confocal microscope and the imaging settings (laser power, gain, 1,024 × 1,024 original resolution, pixel dwell, objective and use of Nyquist zoom) were kept consistent within experiments. For all images, brightness and contrast adjustments were uniformly applied to the entire image. Images were processed and analyzed using FIJI (ImageJ2 v2.3.0/1.53f).

#### Nuclei isolation for snRNA-seq:

Isolation of kidney nuclei was performed as previously described.^36^ Kidneys were dissected out after PBS washout via cardiac perfusion and snap-frozen in liquid nitrogen immediately after dissection. Frozen tissue was stored at −80 °C until subsequent tissue processing. We modified from the TST (INNER cell) nuclei extraction^98^ and the kidney nuclei isolation protocol.^99,100^ The nuclei isolation buffer (NIB) contains 146 mM NaCl, 10 mM Tris-HCl (pH 7.5), 1 mM CaCl_2_, 21 mM MgCl_2_, 0.03% Tween-20, 0.01% BSA, and 1 tablet of cOmplete ULTRA protease inhibitor per 10 mL NIB. The samples were ground for 30 times in 2mL NIB1 (4 mL NIB + 20 μL RNasin Plus + 20 μL SUPERaseIN) with a 2 mL Dounce grinder and a loose pestle, then homogenate was passed through a 200 μm strainer. The homogenate was ground 15 times with a tight pestle, then 2 mL NIB1 was added, and the homogenate was incubated for 5 min on ice. The homogenate was passed through a 40 μm strainer, then centrifuged at 500g for 5 min at 4°C. The pellet was resuspended in 4 mL NIB2 (4 mL NIB + 4 μL RNasin Plus + 4 μL SUPERaseIN) and the suspension was incubated on ice for 5 min, then centrifuged at 500g for 5 min at 4°C. The pellet was resuspended in 1.5 mL of nuclei resuspension buffer (NSB, 10 mL DPBS + 10 μL RNasin Plus), then centrifuged at 500g for 5 min at 4°C. This step was repeated, then the suspension was passed through a 5 μm strainer. The suspension was centrifuged at 500g for 5 min at 4°C, and the pellet was resuspended in 10 mL NSB. The nuclei suspension was mixed with 5μl Vybrant Ruby stain and incubated on ice for 15 min. The nuclei were sorted in 500 μL final resuspension buffer (FSB, 1 mL DPBS with 1% BSA + 5 μL Protector RNase inhibitor) with a low flow rate and pressure to ensure high viability. Two main gates were used. A 561+683 nm emission for the ruby stain and a 488 530/40 nm emission for GFP. Low trigger pulse width was used as singlet discriminator. One hundred thousand nuclei were collected and centrifuged at 500g for 5 min at 4°C. The top supernatant was carefully removed, and 50 μL volume was left to resuspend the nuclei pellet. Ten microliters of nuclei were mixed with 10 μL Trypan Blue and then loaded on the Fuchs-Rosenthal disposable hemocytometer for counting. Nuclei at 700–1,200 nuclei/ μL were directly loaded to 10X chips for Gel Bead-In Emulsions (GEM) generation.

#### snRNA-seq:

snRNA-seq was performed using a Chromium Next GEM Single Cell 3′ Reagent Kit v3.1 (10x Genomics). Single nuclei were partitioned in droplets with single Gel Beads, which contained primers with cell-tagging indexes. Single nucleus suspensions with concentration of at least 300 nuclei/μL were loaded targeting 10,000 nuclei per sample. The resulting cDNA was profiled on a Bioanalyzer NanoChip (Agilent), then used as a template for library preparation according to the reagent kit protocol. The final libraries were profiled on a Tapestation D1000 tape (Agilent) and quantified using real time PCR (Kapa Biosystems) on a StepOnePlus Real Time PCR workstation (Thermo/ABI). Libraries were sequenced on a NovaSeq 6000 (Illumina). FASTQ files were prepared using bcl2fastq (Illumina) and then aligned to a reference genome using Cellranger v.6.1.2 (10x Genomics). Reads were mapped to both exonic and intronic regions to include the pre-mRNA transcriptome. Library preparation and sequencing were done by the OHSU Integrated Genomics Laboratory.

#### snRNA-seq data analysis:

snRNA-seq analysis was performed like what has previously been described^37^ using a Seurat pipeline that included doublet removal, ambient RNA removal, and normalization. Ambient RNA contamination was estimated and removed using SoupX v.1.6.2.^101^ Cells with cleaned-up reads were then subjected to doublet removal using DoubletFinder.^102^ Mitochondrial features were manually removed by excluding all features starting with “*mt-*”. These processing steps were done separately for each sample. Count matrices now only containing singlets were loaded to Seurat v.4.0 for merging and further filtering. Nuclei counts < 1000 or counts > 10,000 were considered low quality nuclei and were filtered out. The filtered Seurat object was then inputted to SCTransform for normalization and variance stabilization.^103^ The normalized count matrix was integrated using the *FindIntegrationAnchors* function. Dimensional reduction analysis was performed using Principal Component Analysis (PCA). The dataset was super-clustered using the functions *FindNeighbors* and *FindClusters* by employing the first 50 PCA dimensions with a 3.0 clustering resolution to manually curate the dataset of likely doublet populations. Clusters with conflicting cell type markers were filtered out (e.g., expression of epithelial markers *Lrp2*, *Slc12a1*, *Slc12a3*, *Nphs1*) before re-clustering with 30 PCA dimensions with 3.0 clustering resolution. For data visualization, dimension, feature, dot, and violin plots were generated using Seurat.

#### Blood collection methods:

For terminal experiments, mice were euthanized via isoflurane overdose and 0.3–0.7 mL whole blood was collected after decapitation. For non-terminal experiments, mice were anesthetized with 3% isoflurane/2% oxygen and < 200 μL whole blood was collected from the retroorbital sinus using a sterile micropipette tip with a fire-polished end. All blood samples were collected into lithium heparin-coated tubes (BD Microtainer #365965). Blood was spun at 1200g for 20 min at 4 °C immediately following collection. Plasma supernatants were collected and stored at −80 °C for up to a month prior to assay, and only freeze-thawed once. Blood was harvested between 2–5pm.

#### Enzyme-linked immunosorbent assays (ELISA):

ELISA was performed using the indicated assays for the following analytes according to the manufacturer’s instructions: renin (LSBio, LS-F508–1), aldosterone (Tecan, RE52301), angiotensin II (Ray Biotech, EIA-ANGII-1), albumin (Abcam, ab108792). The appropriate plasma or urine dilution was empirically determined using a dilution series. Standard curves and extrapolation of sample concentrations were determined using a 4-parameter logistic fit in Prism. Standards and negative controls were run on each plate, and all samples and standards were run in duplicate for each assay. The plasma renin activity assay (Crystal Chem, 80970) utilized a manufacturer’s protocol to detect and quantify Ang I generated from active renin in the sample using provided Ang I standards. Plates were read according to manufacturer’s instructions using the Cytation 3 plate reader (Agilent) with Gen5 software (v2.04).

#### Volume pressure recording measurement of systemic blood pressure:

The CODA High Throughput VPR System (Kent Scientific) was used for all experiments according to published methods.^51^ Briefly, mice were habituated to the appropriately sized rodent restrainer, cuff set, and heated platform daily for 5 days prior to measurements. The same restrainer was used for each mouse for the duration of the experiment (wiping only with deionized water and lint-free tissue) to habituate the mouse to familiar odors and was stored in a sealed plastic bag in between habituations. Only cage mates were tested in parallel to reduce stress. Tail temperature was verified with an infrared thermometer prior to beginning measurements and mice were tested when tail temperature was between 32–35 °C. For each day of measurements (3 days per mouse), 10 acclimation and 20 experimental cycles were performed. Only measurements that passed software quality control (CODA Data Acquisition Software, version 1.06) were analyzed.

#### Urine collection methods:

Mice were lightly scruffed one at a time over sterile hydrophobic LabSand (Braintree Scientific) until they urinated and for no more than 30 sec. This method reliably yielded between 10–100 μL urine per mouse. Urine was immediately collected using a clean micropipette tip and centrifuged at 800g for 10 min at 4 °C. Urine was stored at −80 °C until assessment via ELISA. Urine was harvested at 2pm.

#### Plasma electrolyte measurement:

Blood was collected via cardiac puncture into heparin-coated syringes under isoflurane anesthesia and rapidly transferred into heparinized tubes, then centrifuged at 2,000 g for 5 min at RT. Plasma was separated and stored at −80°C until use. Plasma Na^+^ and K^+^ were determined using a Model 120 dual channel flame photometer (Sherwood Scientific, Cambridge, UK), with samples run in random order.

#### *In vivo* calcium imaging:

In acute, terminal experiments, 3–4-week-old mice were anesthetized continuously (1–2% isoflurane inhaled via nosecone). Juvenile mice were used as previously published due to size constraints preventing the using of mice beyond postnatal day 28. The left kidney was exteriorized through a flank incision. The animal was placed on the stage of the inverted microscope with the exposed kidney mounted in a coverslip-bottomed chamber bathed in normal saline and maintained as described previously.^58–60,104^ Alexa Fluor 680-conjugated albumin (ThermoFisher) and LysoTracker Red (ThermoFisher) were administered i.v. by retro-orbital injections to label the circulating plasma (30 μL bolus of 10 μg/ml solution) and the renin content of JG cells (10 μL bolus of 0.1 mM solution), respectively. The images were acquired using a Leica SP8 DIVE multiphoton confocal fluorescence imaging system with a Leica 25× water-immersion objective (numerical aperture (NA) 1.3) powered by a Chameleon Discovery laser at 970 nm (Coherent, SantaClara, CA) and a DMI8 inverted microscope’s external Leica 4Tune spectral hybrid detectors (emission at 510–530 nm for GCaMP6s, 590–610 nm for LysoTracker Red, 690–710 nm for Alexa Fluor 680; Leica Microsystems, Heidelberg, Germany) as previously published. During imaging, optical sections including the vascular pole and JG cells of superficial glomeruli were selected, and time (xyt) series with 1 frame per 526 ms were recorded over three minutes to measure endogenous JG cell calcium dynamics and oscillations as previously published.^59^

#### Quantification of multiphoton calcium imaging:

The strong, positive signal (GCaMP6s fluorescence and LysoTracker Red fluorescence) and high-resolution multi-photon imaging allowed for easy identification of single JG cell bodies. For the quantification of changes in mean GCaMP6s fluorescence intensity, regions of interest (ROIs) were drawn closely over the total cell body of single cells and the normalized changes in GCaMP6s fluorescence F/F_0_ (fluorescence intensity expressed as a ratio relative to baseline) were measured after the experiment in the defined ROI. For plotting, data were smoothened using the exponential 2^nd^ order method with 10 neighbors (GraphPad Prism).^59^ Diameters of the afferent arteriole were also measured offline. Measurements were made and quantified using the Quantify package of LAS X software (v3.6.0.20104; Leica-Microsystems).

#### Primary cell culture of mouse cells of renin lineage:

Briefly, 4.5 μm tosylactivated Dynabeads (Life Technologies, 14013) were inactivated by overnight incubation at room temperature in sterile 0.2M Tris (pH 8.5) in 1% BSA, collected in a magnetic rack, and washed with divalent-free Hank’s balanced salt solution (HBSS). Mice were euthanized via isoflurane overdose and perfused transcardially with 30 mL of Dynabead solution (200 μL bead suspension in 30 mL HBSS). Both kidneys were rapidly dissected using sterilized tools and minced on ice in 1 mL enzyme solution in a 2 mL tube (100 Kunitz units/mL Type IV DNase I from bovine pancreas, Sigma-Aldrich, D5025) with 1 mg/mL Collagenase A (Sigma-Aldrich, 10103586001) in HBSS) until pieces were < 1 mm. Tissue was transferred using a P1000 pipette with the tip cut off into 25 mL of enzyme solution and digested at 37 °C in a rotisserie oven for 30 minutes. In a biosafety cabinet, digested kidney tissue was passed through a 100 μm mesh nylon strainer into a clean 50 mL conical tube on ice. The tissue was pressed through the strainer using a sterilized blunt glass tool into a fresh 50 mL conical and the strainer was washed with > 15 mL ice-cold HBSS. The samples were centrifuged at 200g for 5 minutes at 4 °C. Supernatant was discarded and pellet was resuspended in 2 mL cold HBSS. Glomeruli were collected on the magnetic rack and washed 3 times with 2 mL HBSS via consecutive collection of glomeruli and careful removal of supernatant using a glass Pasteur pipette. After the final wash, glomeruli were gently resuspended by manual pipetting in 1 mL RPMI complete medium (RPMI 1640 with Glutamax (Life Technologies, 61870036) supplemented with 17% heat-inactivated Fetal Bovine Serum (Life Technologies, 10082147), 100U/mL penicillin/streptomycin (Fisher Scientific, 15–140-122), and 0.1 U/mL recombinant human insulin (Sigma-Aldrich, 91077C-100MG). Glomeruli were plated on tissue culture flasks and incubated at 37 °C with 5% CO_2_. Media was exchanged every other day. Cultures were passaged after 7–10 days post-isolation when cultures became dominated by stellate rather than cobblestone cells. Briefly, cells were washed for 1 minute in PBS, then incubated in 1 mL warm 0.25% Trypsin-EDTA for 30 seconds at 37 °C, followed by 60 seconds with monitoring on a microscope. Digestion was stopped with 2 mL RPMI complete, cultures were gently pipetted to release cells, and cells were plated at a 1:2–1:8 density into a new flask depending on confluence at time of passage. Cells were plated onto 12 mm poly-D-lysine coated glass coverslips (Corning Biocoat #354086) in a 24-well plate. All electrophysiology recordings and RNA isolation were performed at the third passage after isolation, at or just after two weeks *in vitro*.

#### RNA isolation, cDNA preparation, and qPCR:

RNA was isolated from 1 coverslip of cultured cells from two mice using the RNeasy Mini Kit with Turbo DNase (Qiagen, #74104) according to manufacturer’s instructions and quantified via spectrophotometry. cDNA was immediately prepared from RNA using the SuperScript IV Reverse Transcriptase System (Life Technologies, #18091050) according to the manufacturer’s instructions. FastStart Universal probe master mix with Rox (Roche Diagnostics, #4913949001) was used for qPCR. The reactions were run in technical triplicates on the Eco Real-Time PCR instrument (Illumina) using 0.5 μL of the cDNA in a 10 μL volume according to the manufacturer’s instructions. The following PrimeTime qPCR probes (Integrated DNA Technologies) with a FAM reporter dye and a non-fluorescent quencher were used: mouse *Piezo2* (Mm.PT.56a.32860700), mouse *Piezo1* (Mm.PT.58.11048868), and mouse *Hprt* housekeeping gene (Mm.PT.39a.22214828).

#### Electrophysiology:

Whole-cell patch clamp recordings were sampled at 20 kHz and filtered at 2 kHz using an Axopatch 200B amplifier or a Multiclamp 700A amplifier (Molecular Devices) using standard methods to achieve an access resistance of 12.75 ± 0.71 MΩ (n = 117 cells).^25^ Recordings were digitized and stored directly online using a Digidata 1550B or 1550 digitizer (Molecular Devices) and pClamp 10.7 software (Molecular Devices). During experiments, cells were maintained at 21–23 °C in physiological Ringer’s solution (127 mM NaCl, 3 mM KCl, 2.5 mM CaCl_2_, 1 mM MgCl_2_, 10 mM HEPES, 10 mM glucose, pH 7.3, 305 mOsm) and clamped at −80 mV. Electrodes had resistances of 5.98 ± 0.36 MΩ (n = 117 pipettes) when filled with gluconate-based low-chloride intracellular solution: 100 mM K-gluconate, 25 mM KCl, 0.483 mM CaCl_2_, 3 mM MgCl_2_, 10 mM HEPES, 1 mM BAPTA tetrapotassium salt, 4 mM Mg-ATP and 0.4 mM Na-GTP (pH 7.3 with KOH, 295 mOsm). Cells were tested for mechanosensitivity with a fire-polished glass probe. The probe displacement was advanced in increments of 0.5 μm using a computer-controlled piezoelectric stimulator (Physik Instrumente). The recordings were performed in parallel by two experimenters blinded to the genotypes of the cells. Data were combined for analysis by blinded individuals. Recordings from cells isolated from age-matched littermates were performed on the same days. All data were analyzed as previously described using pClamp 10.7.^25^

#### Non-invasive transdermal measurement of glomerular filtration rate (GFR):

GFR was measured using the transdermal system from MediBeacon.^66,105^ Briefly, mouse backs were dehaired with depilatory cream (Nair) on the day prior to measurement. Mice were anesthetized with 2% isoflurane/1% oxygen and placed on a 37 °C heating pad. The transducer was applied to the dehaired flank of the mouse using the supplied adhesive patch, avoiding any pigmented skin regions. Baseline was acquired for 1–3 min. Mice received a bolus injection of 2.5 μL/g b.w. of 30 mg/mL fluorescein isothiocyanate (FITC)-sinistrin (MediBeacon) prepared in sterile PBS and delivered via the retroorbital sinus with a 28G insulin syringe. Measurements were acquired for one-hour post-injection and data were analyzed and fitted offline using the MBLab2 software (MediBeacon, v2.12) according to the manufacturer’s instruction to calculate the *t*_*1/2*_ in minutes. GFR was calculated from *t*_*1/2*_ using the formula for adult C57BL6/J mice:

$$GFR[\mu L/min/100\;g\;b.w]=14616.8[\mu L/100\;g\;b.w.]/t_{1/2\;\mathrm{FITC}-\mathrm{Sinistrin}}[min]$$

#### Urea assay (blood urea nitrogen, BUN):

BUN was measured as for the ELISA assays detailed above in triplicate from plasma samples using the colorimetric QuantiChrom Urea Assay Kit (DIUR-100, BioAssay Systems) using the provided standards.

#### Histology:

Hematoxylin and eosin (H&E) and Periodic-acid Schiff (PAS) staining were performed on formalin-fixed mouse kidney sections by the Sanford Burnham Prebys Histology Core (La Jolla, CA). The slides were analyzed for glomerulopathy, mesangial cell number, mesangial matrix deposition, tubular morphology, and quantity of nuclei in the juxtaglomerular apparatus by an individual blinded to genotypes. In total, sections from n = 4 *Piezo2*^*fl/fl*^*; Pdgfrb*^*WT*^; n = 4 *Piezo2*^*fl/fl*^*; Pdgfrb*^*CreERT2*^ n = 7 *Piezo2*^*fl/fl*^*; Ren*^*WT*^, and n = 4 *Piezo2*^*fl/fl*^*; Ren*^*Cre*^ mice were analyzed. Representative images of kidney cortex were acquired using a Keyence BZ-X710 microscope using brightfield imaging with a 40x objective and the supplied color camera.

Measurement of renin in paraffin-embedded kidney sections: Sections (5 μm) from Bouin’s-fixed, paraffin-embedded kidneys were deparaffinized, rehydrated, and treated with 0.3% hydrogen peroxide in methanol for 30 min at RT. After blocking with 3% BSA + 2% goat serum or horse serum in PBS for 1 h at RT, sections were incubated with a rabbit polyclonal anti-mouse renin antibody (1:500) (generated by R.A.G. and M.S.S.L.)^95^ at 4°C overnight. After washing, sections were incubated with biotinylated secondary antibody: goat anti–rabbit IgG (1:200; Vector Laboratories, BA-1000) at RT for 30 min. Staining was amplified using the Vectastain ABC kit (Vector Laboratories) and developed with 3,3-diaminobenzidine (Sigma). The sections were counterstained with hematoxylin (Sigma), dehydrated, and mounted with Cytoseal XYL (Thermo Fisher Scientific). We determined the juxtaglomerular area index (JGA index) as the number of renin positive JG areas divided by the total number of glomeruli in whole sections for each animal. All the image parameters were kept constant among different samples. Some tubular dilatations were observed in the samples; however, these were observed in mice of both genotypes.

#### Captopril administration:

Water valves were removed from cages and mice were supplied with 400mg/L captopril (Sigma-Aldrich, C4042) in the drinking water, prepared fresh each day in a water bottle. Mice had access to standard chow. After six days of treatment, GFR was measured. On the seventh day, blood was harvested for ELISA.

#### Enzyme activity assays:

Enzyme activities of ACE2, PEP and APA in mouse plasma were measured in black 96-well microtiter plates (Costar, 3915) using 2uL of sample per well as previously described in detail.^83–85^ ACE activity was measured in white 96-well microtiter plates (Costar, 3912) using 1uL of mouse plasma per well.^83,84,106^ Briefly, mouse plasma was added to wells containing a buffer (Tris-buffered saline, pH 7.4 consisting of 19.98 mM Tris, 136 mM NaCl) and the respective substrate. Reactions were run in duplicate (1 of 2 wells constituted a blank). Blank wells contained the same components, but respective specific inhibitors were also added. Each respective enzyme activity was taken as the activity totally inhibitable by the respective specific inhibitor. The measurements were performed with a 100-μL total volume per well. Fluorescence was determined using a microplate fluorescence microplate reader (FLx800, Bio-Tek Instruments, Inc.). Each activity was corrected for the volume of plasma added. Substrates, inhibitors, and excitation/emission wavelengths (in nm) for each enzyme assay were as follows: APA (H-Glu-AMC, amastatin, 380/460), ACE2 (Mca-APK-Dnp, MLN-4760, 320/400), ACE (hippuryl-L-histidyl-L-leucine, captopril, 380/485), PEP (Z-Gly-Pro-AMC, S-17092, 380/460). End concentrations for all substrates and inhibitors were 10^−5^ M, except for captopril (10^−7^ M).

#### A779 administration:

Mice were injected i.p. once daily for seven consecutive days with 0.5 mg/kg A779 (Cayman Chemical, 23396) dissolved in 0.9% NaCl in water.

#### Acute volume loading (hypervolemia) model:

Mice received 100 μL/ gram body weight 0.9% NaCl warmed to 37°C via intraperitoneal injection. Mice were euthanized via isoflurane overdose and terminal blood collection was performed thirty minutes following injection.

#### High- and low-sodium diet experiments (chronic hyper- and hypovolemia):

Mice were maintained under the standard housing conditions described above and fed one of the following three NaCl adjusted diets: Sodium Deficient Diet (Inotiv TD.90228, 0.01–0.02% Na), Control Diet (Inotiv TD.90229, 0.4% Na), High Sodium Diet (Inotiv TD.92012, 3.2% Na). Diets were stored under refrigeration and were replenished every other day to ensure freshness. Mice remained on the diet for two weeks prior to analysis of blood. Weights of animals were monitored throughout the experiment and no significant differences from control were observed (data not shown).

#### Polyethylene glycol (PEG) model of acute hypovolemia:

Mice were induced with 3% isoflurane and injected with 10 μL/ gram body weight 40% w/v PEG-8000 (Sigma-Aldrich, 89510) in sterile 0.9% NaCl subcutaneously using a 28G insulin syringe. Mice were immediately recovered from anesthesia following injection. Food and water were removed from the cage. After six hours, mice were euthanized, and blood was harvested. For the 6-OHDA/indomethacin experiments, 15 minutes after indomethacin injection, mice received either sterile saline or 40% PEG.

#### 6-OHDA and indomethacin administration:

Mice were injected i.p. once daily for four consecutive days with 150 mg/kg 6-OHDA HBr (Sigma-Aldrich, 162957) dissolved in 0.02% ascorbic acid and 0.9% NaCl in water. 24 hours after the final injection, mice were injected i.p. with 5 mg/kg indomethacin (Tocris, 1708) dissolved in 0.01 M sodium carbonate with 1% DMSO in water.

### QUANTIFICATION AND STATISTICAL ANALYSIS

All statistical analyses except for snRNA-seq analysis were performed in Prism v10.1.1(GraphPad). Error bars are defined as the mean ± s.e.m throughout, and individual data points are plotted. For blood pressure data, individual values separated by experimental subject are provided in the Supplementary Figures and nested *t-*tests were performed to account for multiple measurements in the same animal. All tested covariates are reported in the legends. Two-tailed tests were performed wherever applicable. *N*, test statistics, exact *p*-values, and degrees of freedom (d.f.) are indicated where relevant in the figure legends. Normality and/or equal variance were not assumed, and so nonparametric tests were used throughout unless otherwise indicated. Statistical methods for snRNA-seq are detailed separately in the relevant section of the STAR Methods.

No analyses were performed in advance to pre-determine sample size. Sample sizes were based on similar studies in the literature.^18,25,59,64^ No randomization was used. All experiments where genotypes are compared were performed and analyzed while blinded to the genotype of the animals tested. All experiments were independently repeated at least twice, and data were pooled.

### ADDITIONAL RESOURCES

Not applicable.

## Supplementary Material

1 **Figure S1. Features of snRNA-seq dataset, related to** Figure 2. **A**. UMAP projection of mouse kidney stroma split by sample. **B**. Violin plot of nFeature and nCount split by sample. **C**. Violin plot of nFeature and nCount split by stromal cell populations. **D**. Feature plot of markers used to identify distinct kidney stromal cell populations. **E**. Featureplot of markers used to identify distinct kidney stromal cell populations.

2 **Figure S2. Genes corresponding to cell types, related to** Figure 2. **A**. Feature plots of stromal cell sub type marker gene expression in UMAP space. **B**. Violin plots of stromal cell sub type marker gene expression across cell populations.

3 **Figure S3. Additional PIEZO expression and validation of Cre lines, related to** Figures 2 and 3. **A**. smFISH of sectioned C57BL6/J mouse kidney for *Piezo2*, *Ren1*, and counterstained with DAPI, for comparison to **B-C**. **B**. smFISH of sectioned C57BL6/J mouse kidney for *Piezo2*, *Pdgfrb*, and counterstained with DAPI. **C**. smFISH of sectioned C57BL6/J mouse kidney with IHC for *Piezo1*, *Pdgfrb*, and counterstained with DAPI. **D**. Sectioned mouse kidney stained with anti-tdTomato AlexaFluor 647-conjugated nanobody. Asterisk (*) indicates distal convoluted tubule. **E**. smFISH of sectioned human kidney for *PIEZO2*, *PDGFRB*, and counterstained with DAPI. **F**. Sectioned mouse kidney with native tdTomato fluorescence, stained with anti-Renin and anti-PECAM1 antibodies. **G**. Sectioned mouse kidney with native tdTomato fluorescence, stained with anti-NPHS2 and anti-PECAM1 antibodies. **H**. Sectioned mouse kidney with native tdTomato fluorescence, stained with anti-Renin and anti-PECAM1 antibodies. **I**. Sectioned mouse kidney with native tdTomato fluorescence, stained with anti-NPHS2 and anti-PECAM1 antibodies. **J**. Sectioned mouse kidney with native tdTomato fluorescence, stained with anti-NPHS2 and anti-PECAM1 antibodies. **K**. Sectioned mouse kidney with native tdTomato fluorescence, stained with anti-Renin and anti-PECAM1 antibodies. Dotted circles outline renal corpuscles. **L**. Sectioned mouse kidney with native tdTomato fluorescence, stained with anti-Renin antibodies. Asterisks (*) are placed to the immediate left of JGA. Scale bars = 100 μm. Each experiment was repeated on N=2 mice with n=2 slides as technical replicates.

4 **Figure S4. Plasma renin levels in mice, related to** Figure 3. **A**. Plasma renin levels in *Piezo2*^*fl/fl*^*; FoxD1*^*WT*^ versus *Piezo2*^*fl/fl*^*; FoxD1*^*Cre*^ animals (Mann–Whitney: *p = 0.0381, U = 22; n = 9 *FoxD1*^*WT*^ and 11 *FoxD1*^*Cre*^ mice). **B**. Plasma renin levels in *Piezo1*^*fl/fl*^*; Piezo2*^*fl/fl*^*; Pdgfrb*^*WT*^ versus *Piezo1*^*fl/fl*^*; Piezo2*^*fl/fl*^*; Pdgfrb*^*CreERT2*^ animals (Mann–Whitney: **p = 0.0040, U = 2; n = 9 *Pdgfrb*^*WT*^ and 5 *Pdgfrb*^*CreERT2*^ mice). Error bars represent mean ± s.e.m.

5 **Figure S5. Heart rate and individual blood pressure measurements in *Piezo2*^*fl/fl*^*; Pdgfrb*^*CreERT2*^ mice, related to** Figure 3. **A**. Heart rate (beats per minute) measured using the VPR system in *Piezo2*^*fl/fl*^*; Pdgfrb*^*WT*^ versus *Piezo2*^*fl/fl*^*; Pdgfrb*^*CreERT2*^ animals (two-tailed nested *t-*test: = 0.3122, *t* = 1.044, d.f. = 16, n = 10 *Pdgfrb*^*WT*^ and 8 *Pdgfrb*^*CreERT2*^ mice). **B**. Data in **A** replotted to show individual data points per mouse, with *Piezo2*^*fl/fl*^*; Pdgfrb*^*WT*^ in gray and *Piezo2*^*fl/fl*^*; Pdgfrb*^*CreERT2*^ in green. **C**. Systolic blood pressure data from *Piezo2*^*fl/fl*^*; Pdgfrb*^*WT*^ (gray) versus *Piezo2*^*fl/fl*^*; Pdgfrb*^*CreERT2*^ (green) animals replotted from Figure 3G to show all trials from individual mice. **D**. Diastolic blood pressure data from *Piezo2*^*fl/fl*^*; Pdgfrb*^*WT*^ (gray) versus *Piezo2*^*fl/fl*^*; Pdgfrb*^*CreERT2*^ (green) animals replotted from Figure 3G to show all trials from individual mice. **E**. Mean arterial blood pressure data from *Piezo2*^*fl/fl*^*; Pdgfrb*^*WT*^ (gray) versus *Piezo2*^*fl/fl*^*; Pdgfrb*^*CreERT2*^ (green) animals replotted from Figure 3G to show all trials from individual mice. **F**. Summary statistics table. Error bars represent mean ± s.e.m.

6 **Figure S6. Heart rate and individual blood pressure measurements in *Piezo2*^*fl/fl*^*; Ren*^*Cre*^ mice, related to** Figure 3. **A**. Heart rate (beats per minute) measured using the VPR system in *Piezo2*^*fl/fl*^*; Ren*^*WT*^ versus *Piezo2*^*fl/fl*^*; Ren*^*Cre*^ animals (two-tailed nested *t*-test: = 0.2572, *t* = 1.374, d.f. = 17, n = 10 *Ren*^*WT*^ and 9 *Ren*^*Cre*^ mice). **B**. Data in A replotted to show individual data points per mouse, with *Piezo2*^*fl/fl*^*; Ren*^*WT*^ in gray and *Piezo2*^*fl/fl*^*; Ren*^*Cre*^ in magenta. **C**. Systolic blood pressure data from *Piezo2*^*fl/fl*^*; Ren*^*WT*^ (gray) versus *Piezo2*^*fl/fl*^*; Ren*^*Cre*^ (magenta) animals replotted from Figure 3I to show all trials from individual mice. **D**. Diastolic blood pressure data from *Piezo2*^*fl/fl*^*; Ren*^*WT*^ (gray) versus *Piezo2*^*fl/fl*^*; Ren*^*Cre*^ (magenta) animals replotted from Figure 3I to show all trials from individual mice. **E**. Mean arterial blood pressure data from *Piezo2*^*fl/fl*^*; Ren*^*WT*^ (gray) versus *Piezo2*^*fl/fl*^*; Ren*^*Cre*^ (magenta) animals replotted from Figure 3I to show all trials from individual mice. **F**. Summary statistics table. Error bars represent mean ± s.e.m.

7 **Figure S7. Characterization of *Piezo2*^*fl/fl*^*; Ren*^*Cre*^ mice, related to** Figure 3. **A**. Plasma renin activity (PRA) in *Piezo2*^*fl/fl*^*; Ren*^*WT*^ versus *Piezo2*^*fl/fl*^*; Ren*^*Cre*^ animals (Mann–Whitney: ****p < 0.0001, U = 1; n = 12 *Ren*^*WT*^ and 9 *Ren*^*Cre*^ mice). **B**. Potassium levels in *Piezo2*^*fl/fl*^*; Ren*^*WT*^ versus *Piezo2*^*fl/fl*^*; Ren*^*Cre*^ animals (Mann–Whitney: *p* = 0.9378, U = 61.50; n = 14 *Ren*^*WT*^ and 9 *Ren*^*Cre*^ mice). **C**. Sodium levels in *Piezo2*^*fl/fl*^*; Ren*^*WT*^ versus *Piezo2*^*fl/fl*^*; Ren*^*Cre*^ animals (Mann–Whitney: *p* = 0.7459, U = 57.50; n = 14 *Ren*^*WT*^ and 9 *Ren*^*Cre*^ mice). **D**. Cropped image representative of one full kidney section tile-scan each from N = 5 mice from *Piezo2*^*fl/fl*^*; Ren*^*WT*^ mice stained with anti-Renin antibody (scale = 50 μm). **E**. Cropped image representative of one full kidney section tile-scan each from N = 5 mice from *Piezo2*^*fl/fl*^*; Ren*^*Cre*^ mice stained with anti-Renin antibody (scale = 50 μm). **F**. Quantification of ratio of renin+ JGA (JG index) to total glomeruli (Welch’s t-test: *p = 0.0224, t = 2.841; df = 7.788; N = 5 *Ren*^*WT*^ and 5 *Ren*^*CreERT2*^ mice). Error bars represent mean ± s.e.m.

8 **Figure S8. Cells of renin lineage have MA currents and express functional PIEZO2, related to** Figure 4. **A**. Schematic depicting the workflow for the isolation of mouse kidney glomeruli for culture of mesangial and JG cells (created with BioRender.com). **B**. (left) Acutely isolated mouse glomeruli (day in vitro 1); (right) cultures after 8 days of growth showing tdTomato reporter gene positive cells in red (scale bars = 100 μm); (lower) cartoon depicting whole cell recording and mechanical stimulation of cultured cells using the poking assay (created with BioRender.com). **C**. Representative electrophysiology traces (middle, lower) from two tdTomato+ cultured cells isolated from *Piezo2*^*fl/*+^*; Ai9*^*fl/*+^*; Ren*^*Cre*^ mice showing robust poke-evoked currents when held at −80 mV in voltage-clamp mode. Topmost trace indicates probe indentation steps of 0.5 μm. **D**. τ_inactivation_ of MA currents in *Piezo2*^*fl/*+^ versus *Piezo2*^*fl/fl*^*; Ai9*^*fl/*+^*; Ren*^*Cre*^ cells (Mann–Whitney: ***p = 0.0001, U = 231.5; n = 38 *Piezo2*^*fl/*+^and 27 *Piezo2*^*fl/fl*^ cells from 2 mice per genotype). **E**. Current remaining at the end of the indentation phase as a percentage of the peak of the MA currents in *Piezo2*^*fl/*+^ versus *Piezo2*^*fl/fl*^*; Ai9*^*fl/*+^*; Ren*^*Cre*^ cells (Mann–Whitney: ****p < 0.0001, U = 205; n = 38 *Piezo2*^*fl/*+^and 27 *Piezo2*^*fl/fl*^ cells from 2 mice per genotype). **F**. Apparent threshold at which measurable MA currents were elicited from *Piezo2*^*fl/*+^ versus *Piezo2*^*fl/fl*^*; Ai9*^*fl/*+^*; Ren*^*Cre*^ cells (Mann–Whitney: *p* = 0.1232, U = 394; n = 38 *Piezo2*^*fl/*+^and 27 *Piezo2*^*fl/fl*^ cells from 2 mice per genotype). **G**. Maximal inward current (I_max_) during the poke stimulus from *Piezo2*^*fl/*+^ versus *Piezo2*^*fl/fl*^*; Ai9*^*fl/*+^*; Ren*^*Cre*^ cells (Mann–Whitney: *p* = 0.7839, U = 492; n = 38 *Piezo2*^*fl/*+^and 27 *Piezo2*^*fl/fl*^ cells from 2 mice per genotype). **H**. PCR amplification cycles to threshold (C_t_) for indicated genes from cDNA prepared from *Piezo2*^*fl/*+^ cultured mesangial cells at DIV 14–16 (N = cells from 3 mice with technical triplicates). **I**. Maximal inward current (I_max_) during the poke stimulus from *Piezo1*^*fl/fl*^*; Piezo2*^*fl/fl*^*; Pdgfrb*^*WT*^ versus *Piezo1*^*fl/fl*^*; Piezo2*^*fl/fl*^*; Pdgfrb*^*CreERT2*^ cells (Mann–Whitney: ****p < 0.0001, U = 43; n = 25 *Pdgfrb*^*WT*^ and 27 *Pdgfrb*^*CreERT2*^ cells from 2 mice per genotype). k, τ_inactivation_ of MA currents in *Piezo1*^*fl/fl*^*; Piezo2*^*fl/fl*^*; Pdgfrb*^*WT*^ versus *Piezo1*^*fl/fl*^*; Piezo2*^*fl/fl*^*; Pdgfrb*^*CreERT2*^ cells (Mann–Whitney: ****p < 0.0001, U = 47; n = 25 *Pdgfrb*^*WT*^ and 27 *Pdgfrb*^*CreERT2*^ cells from 2 mice per genotype). When currents failed to inactivate and accurate τ_inactivation_ values could not be obtained due to the length of the holding phase of the poking stimulus, an upper limit of τ_inactivation_ of 125 ms given. Each experiment was performed twice, and error bars represent mean ± s.e.m.

9 **Figure S9. GFR measurements, kidney histology, and RAAS activity of PIEZO2-deficient mice, related to** Figure 5. **A**. GFR measured in young (3–6 months) and then mature (11–14 months) *Piezo2*^*fl/fl*^*; Pdgfrb*^*WT*^ and *Piezo2*^*fl/fl*^*; Pdgfrb*^*CreERT2*^ animals. **B**. Comparison of the ratio of (GFR_mature_/GFR_young_) plotted in **A** (Mann–Whitney: *p* > 0.9999, U = 10; n = 5 *Pdgfrb*^*WT*^ and 4 *Pdgfrb*^*CreERT2*^ mice). **C**. Albumin concentration measured from urine of *Piezo2*^*fl/fl*^*; Pdgfrb*^*WT*^ and *Piezo2*^*fl/fl*^*; Pdgfrb*^*CreERT2*^ animals (Mann–Whitney: *p* = 0.0547, U = 49; n = 16 *Pdgfrb*^*WT*^ and 11 *Pdgfrb*^*CreERT2*^ mice). **D**. BUN of *Piezo2*^*fl/fl*^*; Pdgfrb*^*WT*^ and *Piezo2*^*fl/fl*^*; Pdgfrb*^*CreERT2*^ animals (Mann–Whitney: *p* = 0.3599, U = 29; n = 8 *Pdgfrb*^*WT*^ and 10 *Pdgfrb*^*CreERT2*^ mice) E. GFR in *Piezo1*^*fl/fl*^*; Piezo2*^*fl/fl*^*; SNS*^*WT*^ versus *Piezo1*^*fl/fl*^*; Piezo2*^*fl/fl*^*; SNS*^*Cre*^ animals (Mann–Whitney: *p* = 0.9048, U = 9; n = 4 *SNS*^*WT*^ and 5 *SNS*^*Cre*^ mice). **F**. PAS staining of *Piezo2*^*fl/fl*^*; Pdgfrb*^*WT*^ (upper) and *Piezo2*^*fl/fl*^*; Pdgfrb*^*CreERT2*^ (lower) kidney sections. **G**. H&E staining of *Piezo2*^*fl/fl*^*; Pdgfrb*^*WT*^ (upper) and *Piezo2*^*fl/fl*^*; Pdgfrb*^*CreERT2*^ (lower) kidney sections. **H**. PAS staining of *Piezo2*^*fl/fl*^*; Ren*^*WT*^ (upper) and *Piezo2*^*fl/fl*^*; Ren*^*Cre*^ (lower) kidney sections. **I**. H&E staining of *Piezo2*^*fl/fl*^*; Ren*^*WT*^ (upper) and *Piezo2*^*fl/fl*^*; Ren*^*Cre*^ (lower) kidney sections. Images are representative of n = 4 *Piezo2*^*fl/fl*^*; Pdgfrb*^*WT*^; n = 4 *Piezo2*^*fl/fl*^*; Pdgfrb*^*CreERT2*^ n = 7 *Piezo2*^*fl/fl*^*; Ren*^*WT*^, and n = 4 *Piezo2*^*fl/fl*^*; Ren*^*Cre*^ mice (see Methods). Scale bars = 100 μm. **J**. Plasma aldosterone levels in *Piezo2*^*fl/fl*^*; Pdgfrb*^*WT*^ versus *Piezo2*^*fl/fl*^*; Pdgfrb*^*CreERT2*^ animals after seven days of captopril (Mann–Whitney: *p* = 0.7664, U = 45; n = 11 *Pdgfrb*^*WT*^ and 9 *Pdgfrb*^*CreERT2*^ mice). **K**. ACE2 activity in plasma isolated from *Piezo2*^*fl/fl*^*; Pdgfrb*^*WT*^ animals *Piezo2*^*fl/fl*^*; Pdgfrb*^*CreERT2*^ animals (Mann–Whitney: *p* < 0.0001, U = 0; n = 9 *Pdgfrb*^*WT*^ and 8 *Pdgfrb*^*CreERT2*^ mice). **L**. Additional enzyme activity assays from the same samples in **C** (Mann–Whitney tests: *p* > 0.05, n = 9 *Pdgfrb*^*WT*^ and 8 *Pdgfrb*^*CreERT2*^ mice). Each experiment was performed on at least two independent cohorts of mice, and error bars represent mean ± s.e.m.

10 **Figure S10. Plasma renin levels during hypovolemia in PIEZO-deficient mice, related to** Figure 7. **A**. Simple linear regression of matched data points (same mouse) from Figures 3A–C and 7B–D with indicated R squared value (95% C.I. of slope = 0.4478 to 0.6414). **B**. Simple linear regression of matched data points (same mouse) from Figures 3A–B and 7B–C with indicated R squared value (95% C.I. of slope = 33.92 to 49.82). **C**. Plasma renin levels in *Piezo1*^*fl/fl*^*; Piezo2*^*fl/fl*^*; Pdgfrb*^*WT*^ versus *Piezo1*^*fl/fl*^*; Piezo2*^*fl/fl*^*; Pdgfrb*^*CreERT2*^ animals six hours following PEG injection (Mann–Whitney: *p = 0.0315, U = 16; n = 9 *Pdgfrb*^*WT*^ and 9 *Pdgfrb*^*CreERT2*^ mice). **D**. Plasma renin levels in *Piezo1*^*fl/fl*^*; Piezo2*^*fl/fl*^*; SNS*^*WT*^ versus *Piezo1*^*fl/fl*^*; Piezo2*^*fl/fl*^*; SNS*^*Cre*^ animals six hours following PEG injection (Mann–Whitney: *p* = 0.2614, U = 28; n = 12 *SNS*^*WT*^ and 7 *SNS*^*Cre*^ mice). Error bars represent mean ± s.e.m.

11 **Figure S11. PIEZO2 contributes to renin regulation independently of sympathetic and macula densa signaling, related to** Figure 7. **A**. Experimental strategy (created with BioRender.com). **B**. Sectioned mouse kidney stained with anti-tyrosine hydroxylase antibody and DAPI after vehicle treatment. **C**. Sectioned mouse kidney with stained with anti-tyrosine hydroxylase antibody and DAPI after 6-OHDA treatment. Scale bars = 100 μm. Each experiment was repeated on N=2 mice. **D**. Plasma renin levels (two-way ANOVA: **p_*interaction*_ = 0.0059, F(1,29) = 8.831496; Uncorrected Fisher’s LSD (left to right): **** *p* < 0.0001, **** *p* < 0.0001, ****p < 0.0001, ***p = 0.0001; n = 8 *Ren*^*WT*^ saline, 8 *Ren*^*Cre*^ saline, 9 *Ren*^*WT*^ PEG, and 8 *Ren*^*Cre*^ saline mice). **E**. Plasma aldosterone levels in mice from **D**, except for 2 *Ren*^*WT*^ saline samples that were untested due to insufficient sample volume (two-way ANOVA: ***p_*genotype*_ = 0.0004, F(1,27) = 16.17869; Uncorrected Fisher’s LSD (left to right): *p* = 0.4419, **** *p* < 0.0001, ****p < 0.0001, **p = 0.0010; n = 6 *Ren*^*WT*^ saline, 8 *Ren*^*Cre*^ saline, 9 *Ren*^*WT*^ PEG, and 8 *Ren*^*Cre*^ saline mice). Data from **D** and **E** were subjected to a log-transform prior to statistical analysis. Each experiment was performed on at least two independent cohorts of mice, and error bars represent mean ± s.e.m.

12

Supplementary information

No supplementary information is included.

## Figures and Tables

**Key resources table:** 

REAGENT or RESOURCE	SOURCE	IDENTIFIER
Antibodies		
AlexaFluor 647-conjugated FluoTag-X4 anti-RFP single domain antibody	Nanotag	Cat#N0404
chicken anti-GFP antibody	Aves Labs	Cat#GFP-1020
goat anti-chicken Alexa Fluor 488 secondary antibody	Thermo Fisher	Cat#A-11039
rabbit anti-renin antibody	Abcam	Cat#ab212197
rat anti-PECAM1 antibody	Millipore Sigma	Cat#CBL1337-I
rabbit anti-NPHS2 antibody	Abcam	Cat#ab50339
rabbit anti-RFP antibody	Rockland	Cat#600-401-379
goat anti-rabbit AlexaFluor 647 secondary antibody	Thermo Fisher	Cat#A-21245
goat anti-rat AlexaFluor 488 secondary antibody	Thermo Fisher	Cat#A-11006
rabbit polyclonal anti-mouse renin antibody	Nagalakshmi et al.^95^	N/A
biotinylated goat anti–rabbit IgG antibody	Vector Laboratories	Cat#BA-1000
Biological samples		
Human donor kidney tissue sample	Kidney Translational Resource Center at Washington University	N/A
Chemicals, peptides, and recombinant proteins		
Tamoxifen	Sigma-Aldrich	Cat#T5648
TO-PRO-3 Iodide	Thermo Fisher	Cat#T3605
Alexa Fluor 680-conjugated albumin	Thermo Fisher	Cat#A34787
LysoTracker Red	Thermo Fisher	Cat#L7528
Recombinant human insulin	Sigma-Aldrich	Cat#91077C-100MG
FITC-sinistrin	MediBeacon	Cat#NC1570801
A-779	Cayman Chemical	Cat#23396
PEG-8000	Sigma-Aldrich	Cat#89510
6-OHDA HBr	Sigma-Aldrich	Cat#162957
Indomethacin	Tocris	Cat#1708
Type IV DNase I from bovine pancreas	Sigma-Aldrich	Cat#D5025
Collagenase A	Sigma-Aldrich	Cat#10103586001
H-Glu-AMC	Bachem	Cat#4002702
Amastatin	Sigma-Aldrich	Cat#A1276
Mca-APK-Dnp	Anaspec	Cat#AS-60757
MLN-4760	Millennium Pharmaceuticals	N/A
hippuryl-L-histidyl-L-leucine	Sigma-Aldrich	Cat#H1635
Captopril	Sigma-Aldrich	Cat#C4042
Z-Gly-Pro-AMC	Bachem	Cat#4002518
S-17092	Sigma-Aldrich	Cat#SML0181
Critical commercial assays		
RNAscope Multiplex Fluorescent Reagent Kit V2	ACDBio	Cat#323100
Chromium Next GEM Single Cell 3′ Reagent Kit v3.1	10x Genomics	Cat#PN-1000128
Renin ELISA kit	LSBio	Cat#LS-F508-1
Aldosterone ELISA kit	Tecan	Cat#RE52301
Angiotensin II ELISA kit	Ray Biotech	Cat#EIA-ANGII-1
Albumin ELISA kit	Abcam	Cat#ab108792
Plasma Renin activity assay	Crystal Chem	Cat#80970
RNeasy Mini Kit with Turbo DNase	Qiagen	Cat#74104
SuperScript IV Reverse Transcriptase System	Thermo Fisher	Cat#18091050
FastStart Universal probe master mix with Rox	Roche Diagnostics	Cat#4913949001
QuantiChrom Urea Assay Kit	BioAssay Systems	Cat#DIUR-100
Deposited data		
Single nucleus RNA-seq data	This paper	GSE280628
Experimental models: Organisms/strains		
Mouse: *B6;129-Piezo1*^*tm1.1Apat/*^*J*	Jackson Laboratories	JAX:029214
Mouse: *B6(SJL)-Piezo2*^*tm1.1(cre)Apat*^*/J*	Jackson Laboratories	JAX:027720
Mouse: *B6.Cg-Piezo1*^*tm2.1Apat*^*/J*	Jackson Laboratories	JAX:029213
Mouse: *B6(SJL)-Piezo2*^*tm2.2Apat*^*/J*	Jackson Laboratories	JAX:002014
Mouse: *B6.Cg-Gt(ROSA)26Sor*^*tm9(CAG-tdTomato)Hze*^*/J*	Jackson Laboratories	JAX:007909
Mouse: *B6.Cg-Gt(ROSA)26Sor*^*tm14(CAG-tdTomato)Hze*^*/J*	Jackson Laboratories	JAX:007914
Mouse: *B6.Cg-Pdgfrb*^*tm1.1(cre/ERT2)Csln*^*/J*	Jackson Laboratories	JAX:030201
Mouse: *B6;129S4-Foxd1*^*tm1(GFP/cre)Amc*^*/J*	Jackson Laboratories	JAX:012463
Mouse: B6J.Cg-*Gt(ROSA)26Sor*^*tm96(CAG-GCaMP6s)Hze*^/MwarJ	Jackson Laboratories	JAX:028866
Mouse: *Ren1c*^*Cre*^	Pippin et al.^47^	N/A
Mouse: *Ren1c*^*CreER*^	Pippin et al.^47^	N/A
Mouse: *Tg(Scn10a-cre)*^*1Rkun*^	Gift from R. Kuner	MGI:3042874
Mouse: *B6.Cg-Tg(Pdgfrb-cre/ERT2)6096Rha/J*	Jackson Laboratories	JAX:029684
Mouse: B6;129-Gt(ROSA)26Sor^tm5(CAG-Sun1/sfGFP)Nat^/J	Jackson Laboratories	JAX:021039
Oligonucleotides		
PrimeTime qPCR probe against mouse *Piezo2*	Integrated DNA Technologies	Cat# Mm.PT.56a.32860700
PrimeTime qPCR probe against mouse *Piezo1*	Integrated DNA Technologies	Cat# Mm.PT.58.11048868
PrimeTime qPCR probe against mouse *Hprt*	Integrated DNA Technologies	Cat# Mm.PT.39a.22214828
Software and algorithms		
Prism v10.1.1	GraphPad	RRID:SCR_002798
FIJI (ImageJ2 v2.3.0/1.53f)	http://fiji.sc	RRID:SCR_002285
SoupX v.1.6.2	Young et al.^101^	RRID:SCR_019193
Seurat v.4.0	http://seurat.r-forge.r-project.org	RRID:SCR_007322
Cellranger v.6.1.2	10x Genomics	RRID:SCR_017344
Gen5 v2.04	Biotek	RRID:SCR_017317
CODA Data Acquisition Software v1.06	Kent Scientific	RRID:SCR_018585
LAS X v3.6.0.20104	Leica Microsystems	RRID:SCR_013673
pCIamp v10.7	Molecular Devices	RRID:SCR_011323
MBLab2 v2.12	MediBeacon	N/A
Custom scripts for snRNA-seq analysis and plotting	This paper	DOI: 10.5281/zenodo.17487554
Other		
Mm-*Piezo1*	ACDBio	Cat#400181
Mm-*Piezo2*	ACDBio	Cat#400191
Mm-*Piezo2*-E43-E45	ACDBio	Cat#439971
Mm-*Ren1*	ACDBio	Cat#433461
Mm-*Pdgfrb*	ACDBio	Cat#411381
Mm-*Pecam1*	ACDBio	Cat#316721
Hs-*PIEZO1*	ACDBio	Cat#485101
Hs-*PIEZO2*	ACDBio	Cat#449951
Hs-*PDGFRB*	ACDBio	Cat#548991
3-plex Negative Control Probe	ACDBio	Cat#320871
OHSU Integrated Genomics Laboratory	Oregon Health & Science University	RRID:SCR_022651
4.5 μm tosylactivated dynabeads	Thermo Fisher	Cat#14013
Sodium Deficient Diet	Inotiv	Cat#TD.90228
Control Diet	Inotiv	Cat#TD.90229
High Sodium Diet	Inotiv	Cat#TD.92012
12 mm poly-D-lysine coated glass coverslips	Corning	Cat#354086
Microtainer lithium heparin-coated tubes	BD	Cat#365965
SlowFade Diamond mounting medium	Thermo Fisher	Cat#S36967

## Highlights:

- PIEZO2 regulates renin levels in mice.
- Calcium oscillations in renin cells depend on PIEZO2.
- PIEZO2 regulates RAAS induction during blood volume challenge.
- PIEZO2 functions in parallel with other regulators of renin and RAAS.
