# Supplementary figures and images for: Renal PIEZO2 is an essential regulator of renin
Source: Cell. Author manuscript; Available in PMC 2025 Dec 11. (PMC12695021; doi:10.1016/j.cell.2025.11.013)

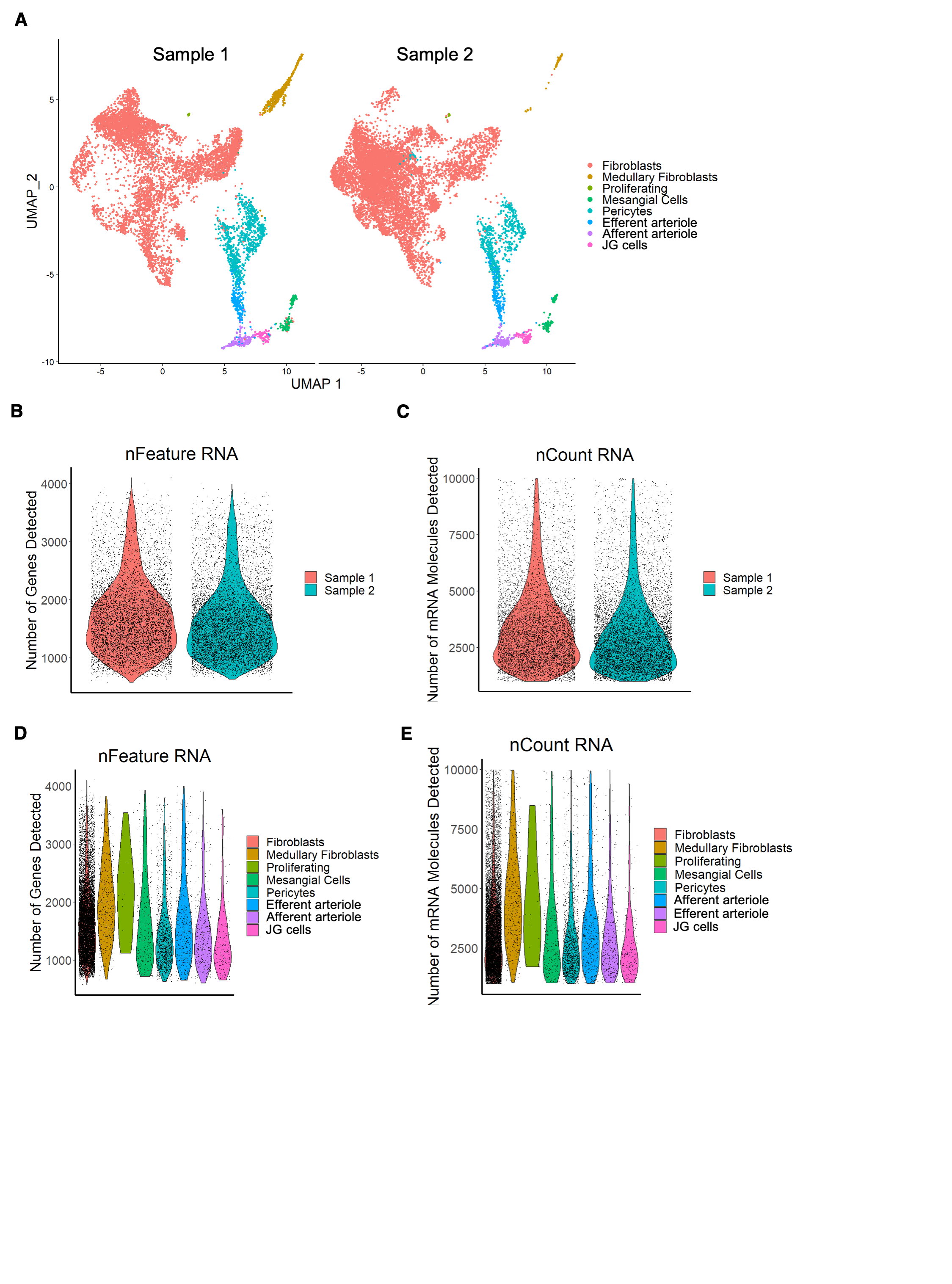

Supplement: 1 — Figure S1. Features of snRNA-seq dataset, related to Figure 2. A. UMAP projection of mouse kidney stroma split by sample. B. Violin plot of nFeature and nCount split by sample. C. Violin plot of nFeature and nCount split by stromal cell populations. D. Feature plot of markers used to identify distinct kidney stromal cell populations. E. Featureplot of markers used to identify distinct kidney stromal cell populations. [file NIHMS2122356-supplement-1.tiff]

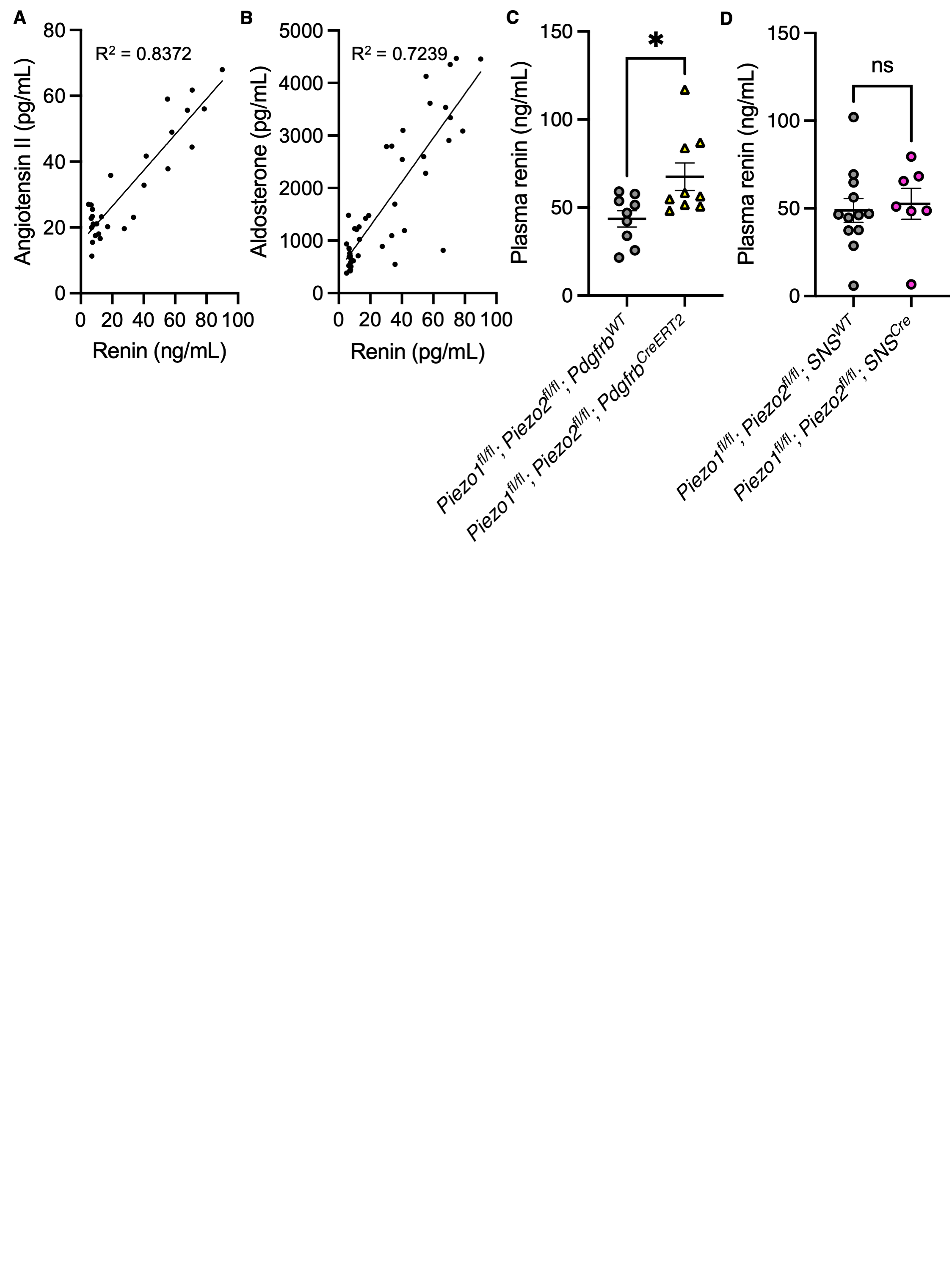

Supplement: 2 — Figure S2. Genes corresponding to cell types, related to Figure 2. A. Feature plots of stromal cell sub type marker gene expression in UMAP space. B. Violin plots of stromal cell sub type marker gene expression across cell populations. [file NIHMS2122356-supplement-2.tiff]

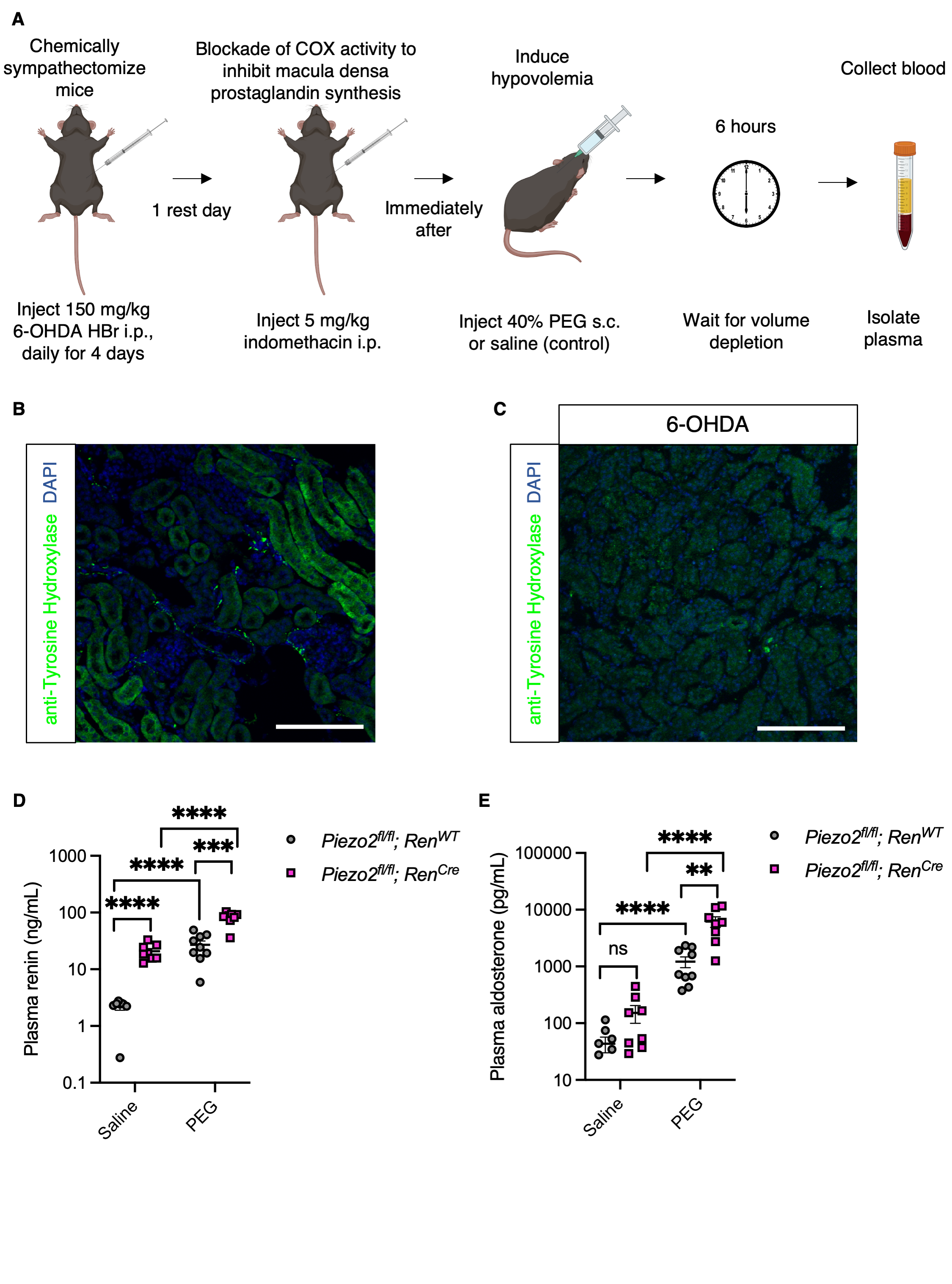

Supplement: 3 — Figure S3. Additional PIEZO expression and validation of Cre lines, related to Figures 2 and 3. A. smFISH of sectioned C57BL6/J mouse kidney for Piezo2, Ren1, and counterstained with DAPI, for comparison to B-C. B. smFISH of sectioned C57BL6/J mouse kidney for Piezo2, Pdgfrb, and counterstained with DAPI. C. smFISH of sectioned C57BL6/J mouse kidney with IHC for Piezo1, Pdgfrb, and counterstained with DAPI. D. Sectioned mouse kidney stained with anti-tdTomato AlexaFluor 647-conjugated nanobody. Asterisk (*) indicates distal convoluted tubule. E. smFISH of sectioned human kidney for PIEZO2, PDGFRB, and counterstained with DAPI. F. Sectioned mouse kidney with native tdTomato fluorescence, stained with anti-Renin and anti-PECAM1 antibodies. G. Sectioned mouse kidney with native tdTomato fluorescence, stained with anti-NPHS2 and anti-PECAM1 antibodies. H. Sectioned mouse kidney with native tdTomato fluorescence, stained with anti-Renin and anti-PECAM1 antibodies. I. Sectioned mouse kidney with native tdTomato fluorescence, stained with anti-NPHS2 and anti-PECAM1 antibodies. J. Sectioned mouse kidney with native tdTomato fluorescence, stained with anti-NPHS2 and anti-PECAM1 antibodies. K. Sectioned mouse kidney with native tdTomato fluorescence, stained with anti-Renin and anti-PECAM1 antibodies. Dotted circles outline renal corpuscles. L. Sectioned mouse kidney with native tdTomato fluorescence, stained with anti-Renin antibodies. Asterisks (*) are placed to the immediate left of JGA. Scale bars = 100 μm. Each experiment was repeated on N=2 mice with n=2 slides as technical replicates. [file NIHMS2122356-supplement-3.tiff]

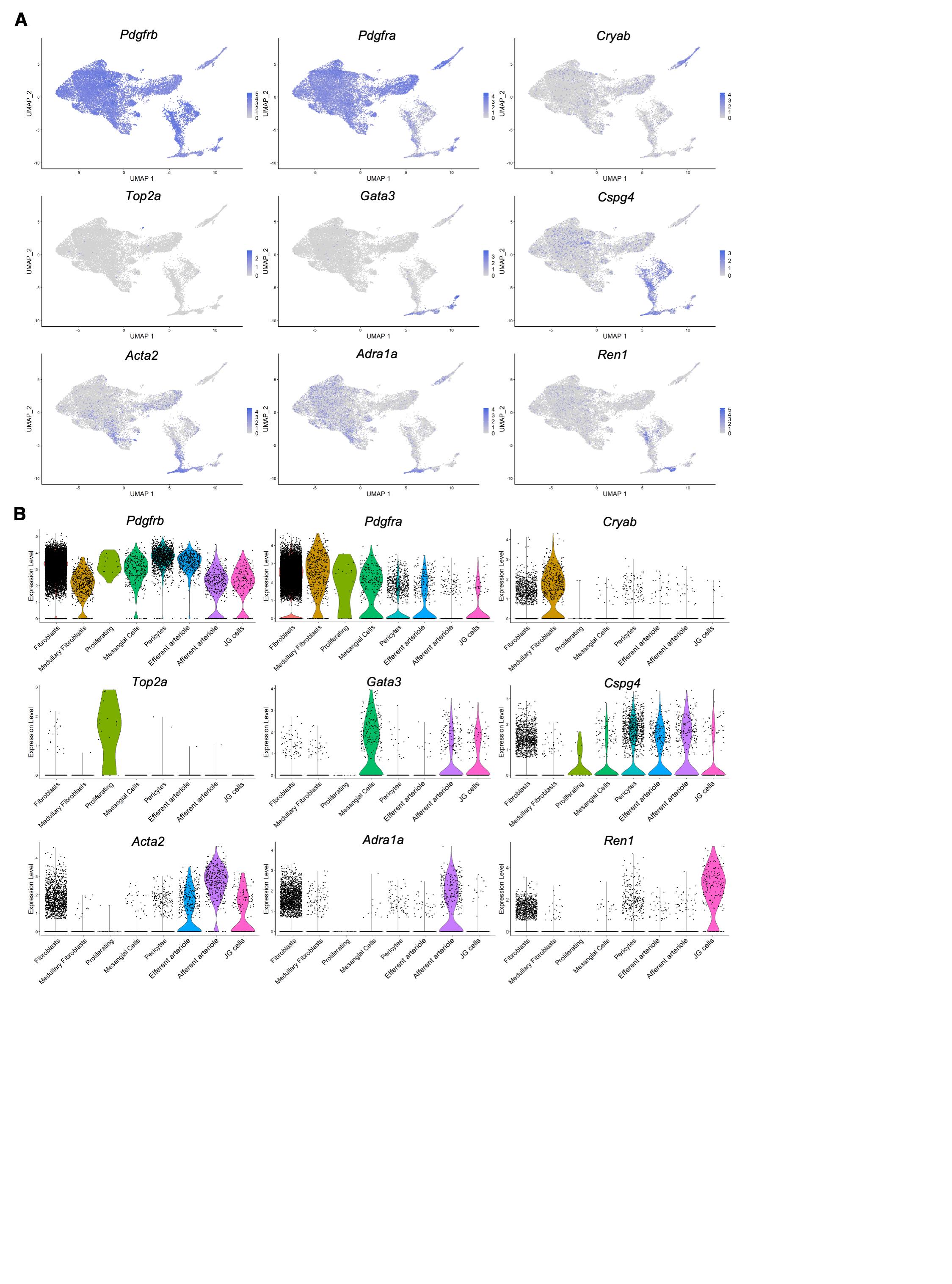

Supplement: 4 — Figure S4. Plasma renin levels in mice, related to Figure 3. A. Plasma renin levels in Piezo2fl/fl; FoxD1WT versus Piezo2fl/fl; FoxD1Cre animals (Mann–Whitney: *p = 0.0381, U = 22; n = 9 FoxD1WT and 11 FoxD1Cre mice). B. Plasma renin levels in Piezo1fl/fl; Piezo2fl/fl; PdgfrbWT versus Piezo1fl/fl; Piezo2fl/fl; PdgfrbCreERT2 animals (Mann–Whitney: **p = 0.0040, U = 2; n = 9 PdgfrbWT and 5 PdgfrbCreERT2 mice). Error bars represent mean ± s.e.m. [file NIHMS2122356-supplement-4.tiff]

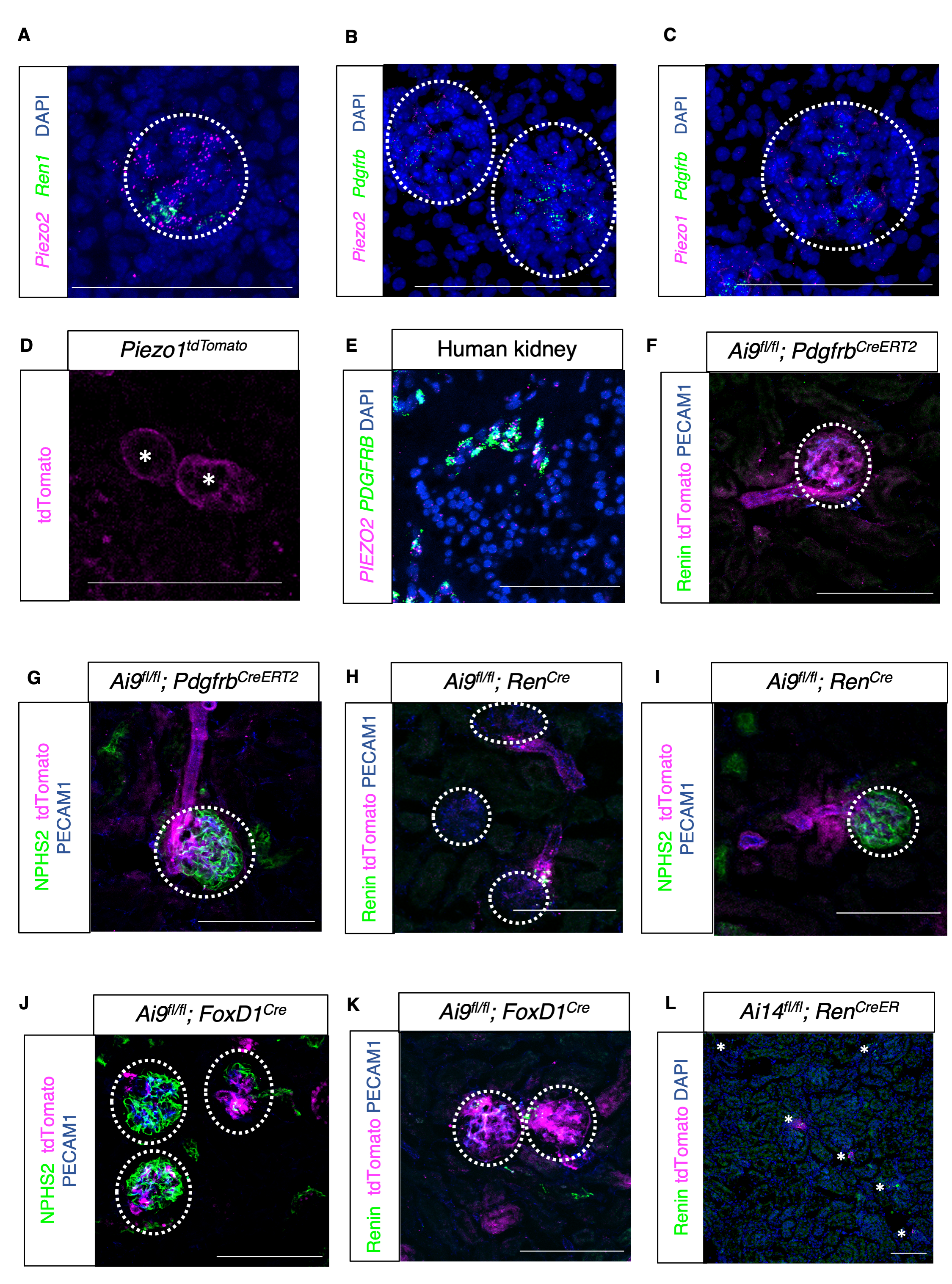

Supplement: 5 — Figure S5. Heart rate and individual blood pressure measurements in Piezo2fl/fl; PdgfrbCreERT2 mice, related to Figure 3. A. Heart rate (beats per minute) measured using the VPR system in Piezo2fl/fl; PdgfrbWT versus Piezo2fl/fl; PdgfrbCreERT2 animals (two-tailed nested t-test: = 0.3122, t = 1.044, d.f. = 16, n = 10 PdgfrbWT and 8 PdgfrbCreERT2 mice). B. Data in A replotted to show individual data points per mouse, with Piezo2fl/fl; PdgfrbWT in gray and Piezo2fl/fl; PdgfrbCreERT2 in green. C. Systolic blood pressure data from Piezo2fl/fl; PdgfrbWT (gray) versus Piezo2fl/fl; PdgfrbCreERT2 (green) animals replotted from Figure 3G to show all trials from individual mice. D. Diastolic blood pressure data from Piezo2fl/fl; PdgfrbWT (gray) versus Piezo2fl/fl; PdgfrbCreERT2 (green) animals replotted from Figure 3G to show all trials from individual mice. E. Mean arterial blood pressure data from Piezo2fl/fl; PdgfrbWT (gray) versus Piezo2fl/fl; PdgfrbCreERT2 (green) animals replotted from Figure 3G to show all trials from individual mice. F. Summary statistics table. Error bars represent mean ± s.e.m. [file NIHMS2122356-supplement-5.tiff]

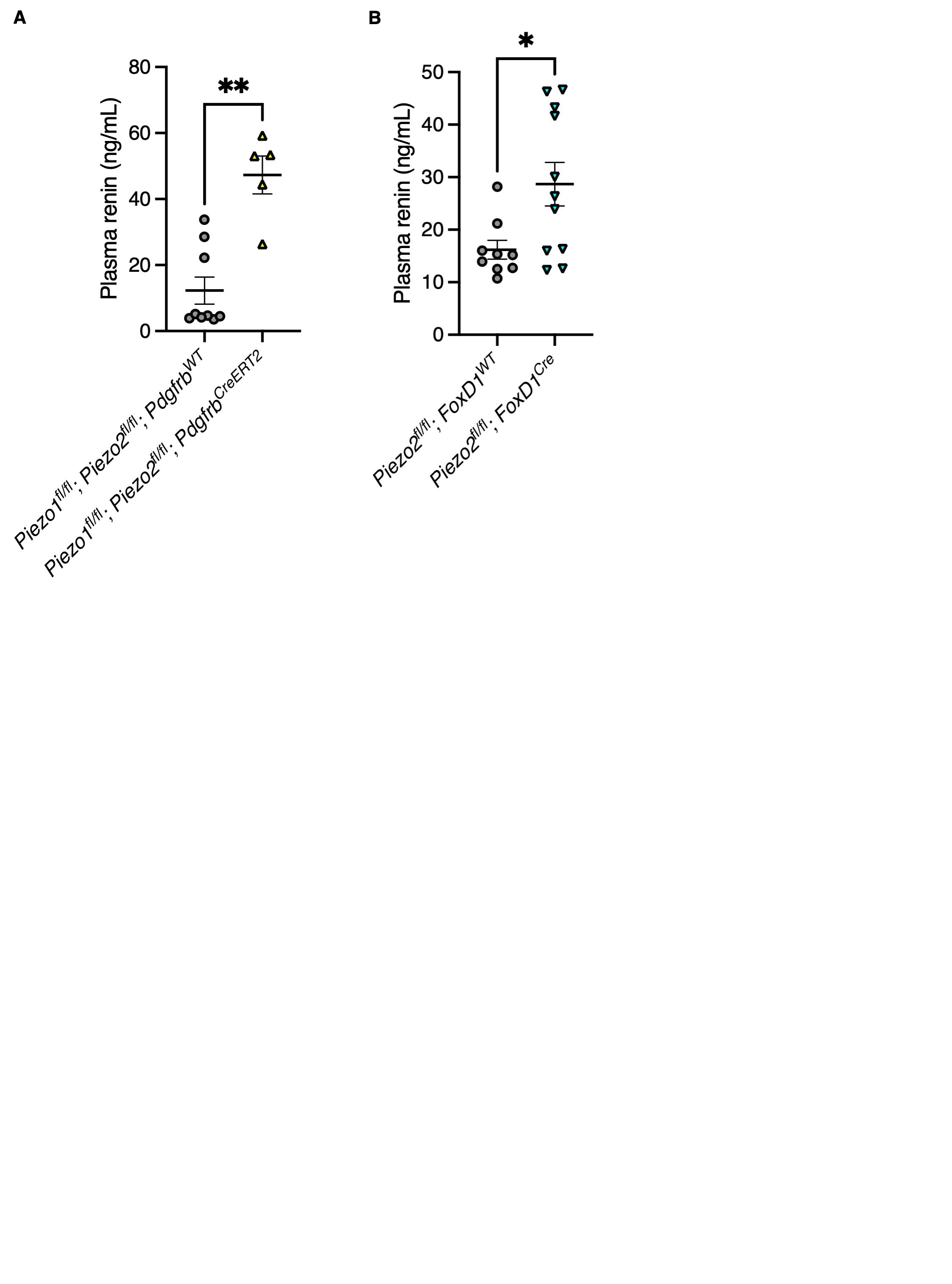

Supplement: 6 — Figure S6. Heart rate and individual blood pressure measurements in Piezo2fl/fl; RenCre mice, related to Figure 3. A. Heart rate (beats per minute) measured using the VPR system in Piezo2fl/fl; RenWT versus Piezo2fl/fl; RenCre animals (two-tailed nested t-test: = 0.2572, t = 1.374, d.f. = 17, n = 10 RenWT and 9 RenCre mice). B. Data in A replotted to show individual data points per mouse, with Piezo2fl/fl; RenWT in gray and Piezo2fl/fl; RenCre in magenta. C. Systolic blood pressure data from Piezo2fl/fl; RenWT (gray) versus Piezo2fl/fl; RenCre (magenta) animals replotted from Figure 3I to show all trials from individual mice. D. Diastolic blood pressure data from Piezo2fl/fl; RenWT (gray) versus Piezo2fl/fl; RenCre (magenta) animals replotted from Figure 3I to show all trials from individual mice. E. Mean arterial blood pressure data from Piezo2fl/fl; RenWT (gray) versus Piezo2fl/fl; RenCre (magenta) animals replotted from Figure 3I to show all trials from individual mice. F. Summary statistics table. Error bars represent mean ± s.e.m. [file NIHMS2122356-supplement-6.tiff]

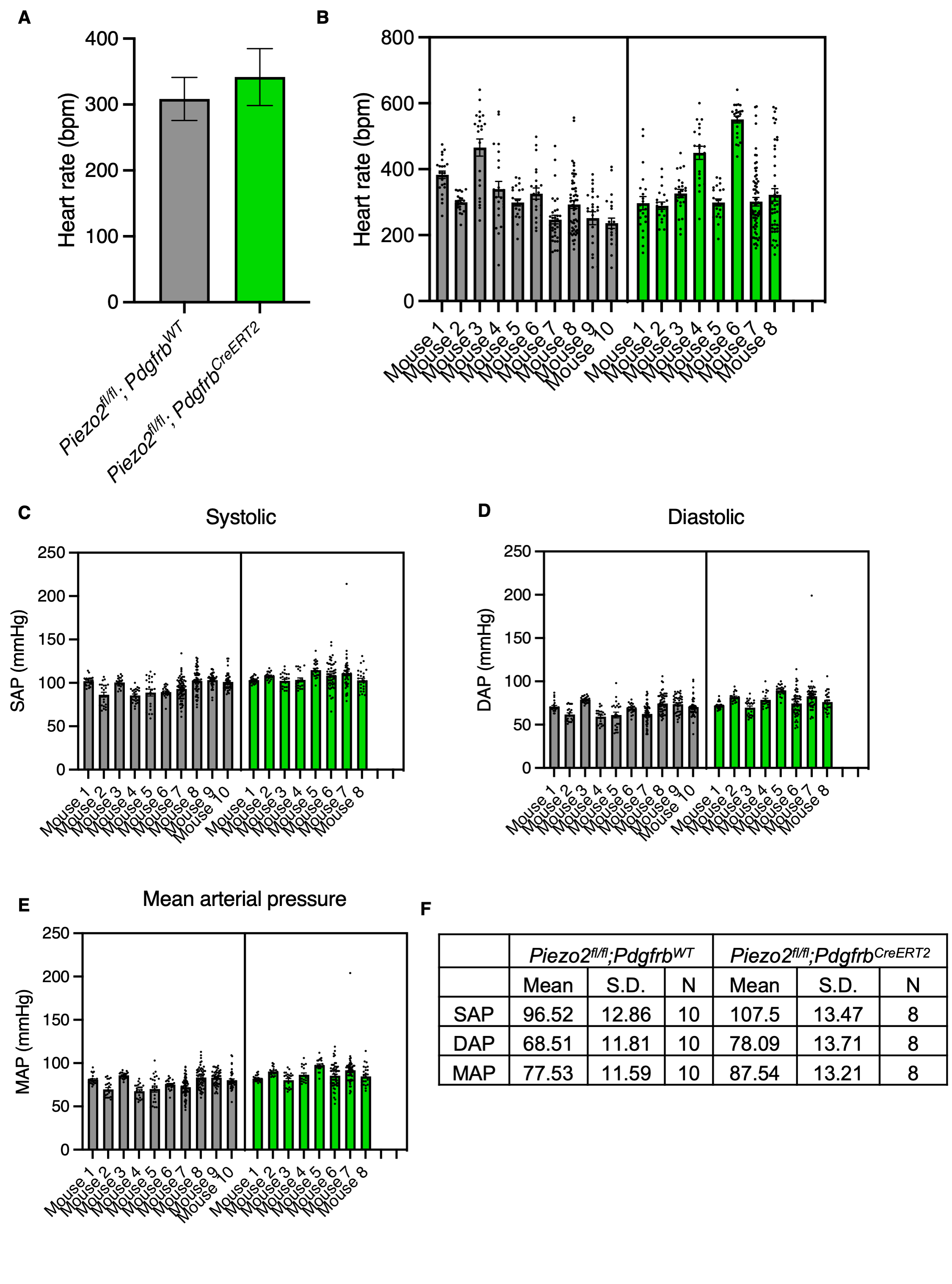

Supplement: 7 — Figure S7. Characterization of Piezo2fl/fl; RenCre mice, related to Figure 3. A. Plasma renin activity (PRA) in Piezo2fl/fl; RenWT versus Piezo2fl/fl; RenCre animals (Mann–Whitney: ****p < 0.0001, U = 1; n = 12 RenWT and 9 RenCre mice). B. Potassium levels in Piezo2fl/fl; RenWT versus Piezo2fl/fl; RenCre animals (Mann–Whitney: p = 0.9378, U = 61.50; n = 14 RenWT and 9 RenCre mice). C. Sodium levels in Piezo2fl/fl; RenWT versus Piezo2fl/fl; RenCre animals (Mann–Whitney: p = 0.7459, U = 57.50; n = 14 RenWT and 9 RenCre mice). D. Cropped image representative of one full kidney section tile-scan each from N = 5 mice from Piezo2fl/fl; RenWT mice stained with anti-Renin antibody (scale = 50 μm). E. Cropped image representative of one full kidney section tile-scan each from N = 5 mice from Piezo2fl/fl; RenCre mice stained with anti-Renin antibody (scale = 50 μm). F. Quantification of ratio of renin+ JGA (JG index) to total glomeruli (Welch’s t-test: *p = 0.0224, t = 2.841; df = 7.788; N = 5 RenWT and 5 RenCreERT2 mice). Error bars represent mean ± s.e.m. [file NIHMS2122356-supplement-7.tiff]

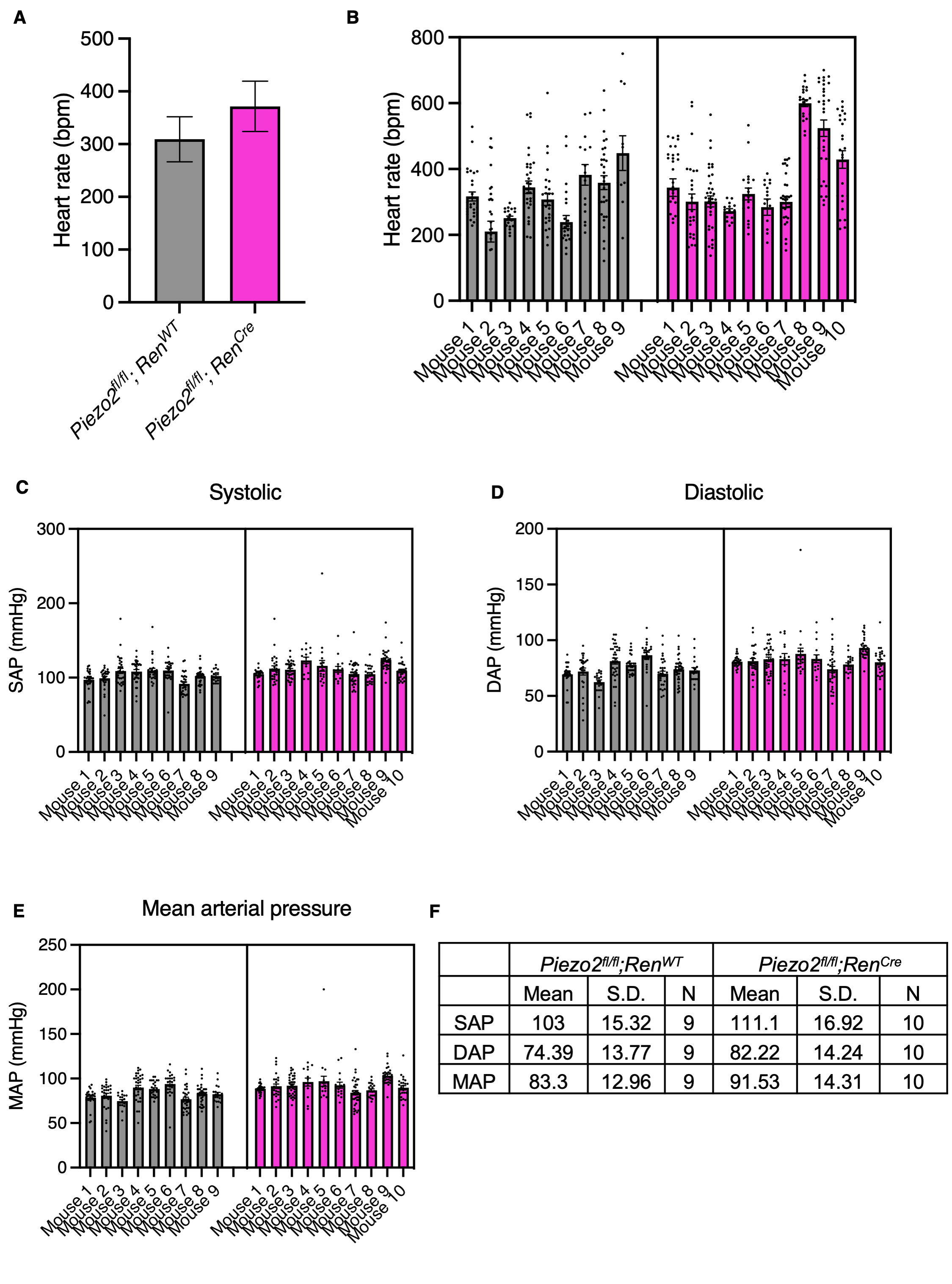

Supplement: 8 — Figure S8. Cells of renin lineage have MA currents and express functional PIEZO2, related to Figure 4. A. Schematic depicting the workflow for the isolation of mouse kidney glomeruli for culture of mesangial and JG cells (created with BioRender.com). B. (left) Acutely isolated mouse glomeruli (day in vitro 1); (right) cultures after 8 days of growth showing tdTomato reporter gene positive cells in red (scale bars = 100 μm); (lower) cartoon depicting whole cell recording and mechanical stimulation of cultured cells using the poking assay (created with BioRender.com). C. Representative electrophysiology traces (middle, lower) from two tdTomato+ cultured cells isolated from Piezo2fl/+; Ai9fl/+; RenCre mice showing robust poke-evoked currents when held at −80 mV in voltage-clamp mode. Topmost trace indicates probe indentation steps of 0.5 μm. D. τinactivation of MA currents in Piezo2fl/+ versus Piezo2fl/fl; Ai9fl/+; RenCre cells (Mann–Whitney: ***p = 0.0001, U = 231.5; n = 38 Piezo2fl/+and 27 Piezo2fl/fl cells from 2 mice per genotype). E. Current remaining at the end of the indentation phase as a percentage of the peak of the MA currents in Piezo2fl/+ versus Piezo2fl/fl; Ai9fl/+; RenCre cells (Mann–Whitney: ****p < 0.0001, U = 205; n = 38 Piezo2fl/+and 27 Piezo2fl/fl cells from 2 mice per genotype). F. Apparent threshold at which measurable MA currents were elicited from Piezo2fl/+ versus Piezo2fl/fl; Ai9fl/+; RenCre cells (Mann–Whitney: p = 0.1232, U = 394; n = 38 Piezo2fl/+and 27 Piezo2fl/fl cells from 2 mice per genotype). G. Maximal inward current (Imax) during the poke stimulus from Piezo2fl/+ versus Piezo2fl/fl; Ai9fl/+; RenCre cells (Mann–Whitney: p = 0.7839, U = 492; n = 38 Piezo2fl/+and 27 Piezo2fl/fl cells from 2 mice per genotype). H. PCR amplification cycles to threshold (Ct) for indicated genes from cDNA prepared from Piezo2fl/+ cultured mesangial cells at DIV 14–16 (N = cells from 3 mice with technical triplicates). I. Maximal inward current (Imax) during [file NIHMS2122356-supplement-8.tiff]

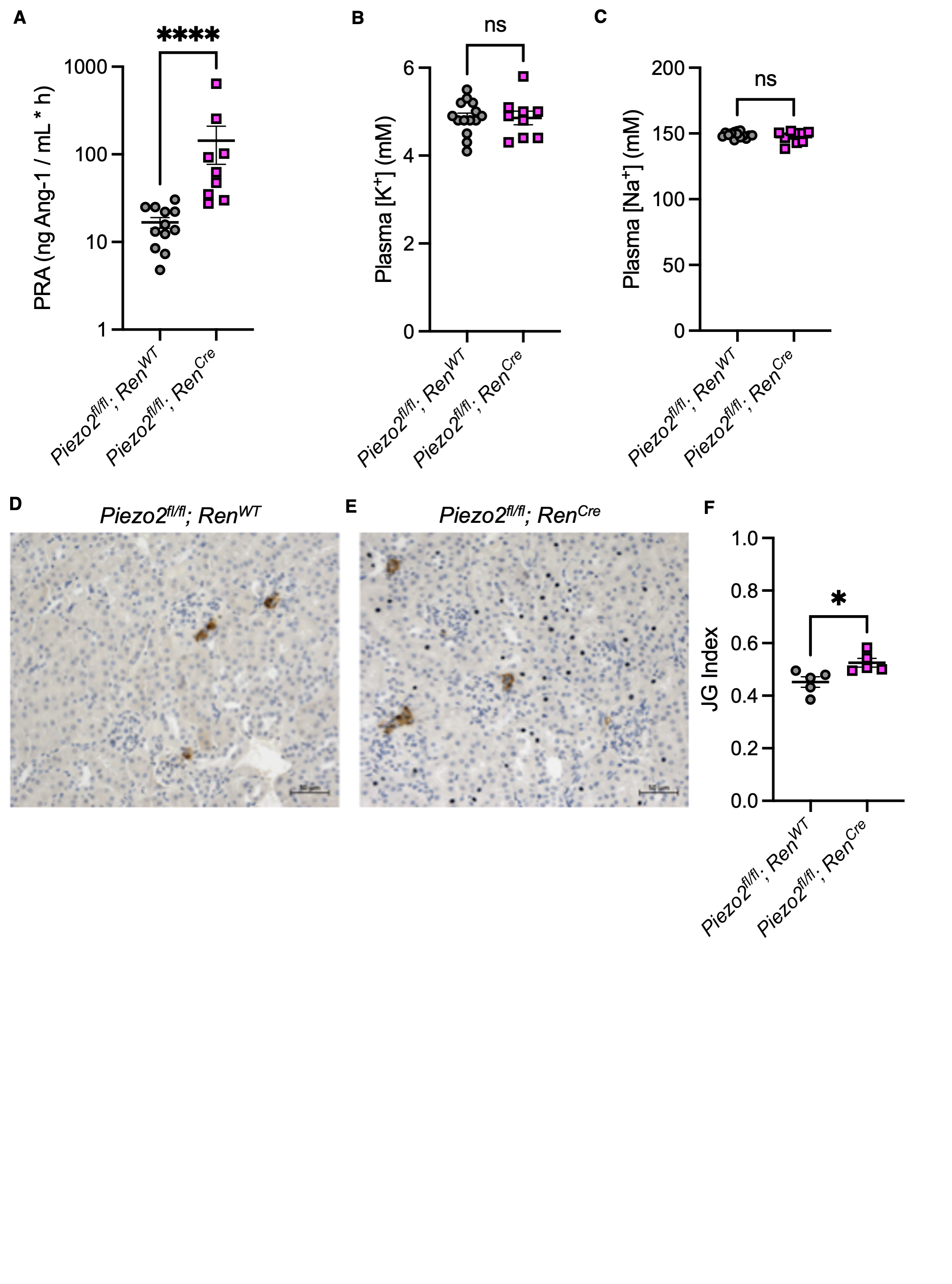

Supplement: 9 — Figure S9. GFR measurements, kidney histology, and RAAS activity of PIEZO2-deficient mice, related to Figure 5. A. GFR measured in young (3–6 months) and then mature (11–14 months) Piezo2fl/fl; PdgfrbWT and Piezo2fl/fl; PdgfrbCreERT2 animals. B. Comparison of the ratio of (GFRmature/GFRyoung) plotted in A (Mann–Whitney: p > 0.9999, U = 10; n = 5 PdgfrbWT and 4 PdgfrbCreERT2 mice). C. Albumin concentration measured from urine of Piezo2fl/fl; PdgfrbWT and Piezo2fl/fl; PdgfrbCreERT2 animals (Mann–Whitney: p = 0.0547, U = 49; n = 16 PdgfrbWT and 11 PdgfrbCreERT2 mice). D. BUN of Piezo2fl/fl; PdgfrbWT and Piezo2fl/fl; PdgfrbCreERT2 animals (Mann–Whitney: p = 0.3599, U = 29; n = 8 PdgfrbWT and 10 PdgfrbCreERT2 mice) E. GFR in Piezo1fl/fl; Piezo2fl/fl; SNSWT versus Piezo1fl/fl; Piezo2fl/fl; SNSCre animals (Mann–Whitney: p = 0.9048, U = 9; n = 4 SNSWT and 5 SNSCre mice). F. PAS staining of Piezo2fl/fl; PdgfrbWT (upper) and Piezo2fl/fl; PdgfrbCreERT2 (lower) kidney sections. G. H&E staining of Piezo2fl/fl; PdgfrbWT (upper) and Piezo2fl/fl; PdgfrbCreERT2 (lower) kidney sections. H. PAS staining of Piezo2fl/fl; RenWT (upper) and Piezo2fl/fl; RenCre (lower) kidney sections. I. H&E staining of Piezo2fl/fl; RenWT (upper) and Piezo2fl/fl; RenCre (lower) kidney sections. Images are representative of n = 4 Piezo2fl/fl; PdgfrbWT; n = 4 Piezo2fl/fl; PdgfrbCreERT2 n = 7 Piezo2fl/fl; RenWT, and n = 4 Piezo2fl/fl; RenCre mice (see Methods). Scale bars = 100 μm. J. Plasma aldosterone levels in Piezo2fl/fl; PdgfrbWT versus Piezo2fl/fl; PdgfrbCreERT2 animals after seven days of captopril (Mann–Whitney: p = 0.7664, U = 45; n = 11 PdgfrbWT and 9 PdgfrbCreERT2 mice). K. ACE2 activity in plasma isolated from Piezo2fl/fl; PdgfrbWT animals Piezo2fl/fl; PdgfrbCreERT2 animals (Mann–Whitney: p < 0.0001, U = 0; n = 9 PdgfrbWT and 8 PdgfrbCreERT2 mice). L. Additional enzyme activity assays from the same samples in C (Mann–Whitney tests: p > 0.05, n = 9 PdgfrbWT and 8 PdgfrbCreERT2 mice). Each experime [file NIHMS2122356-supplement-9.tiff]

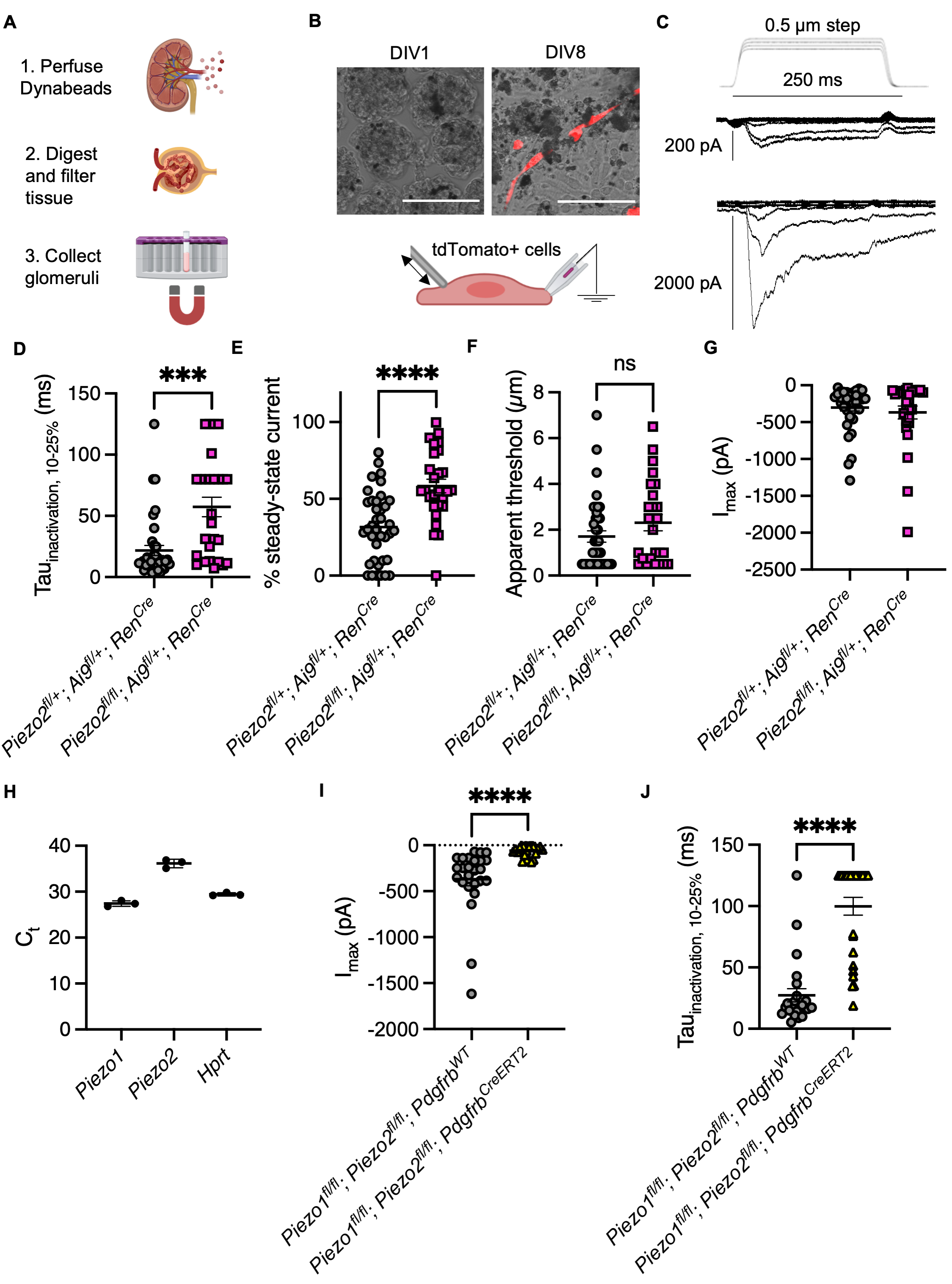

Supplement: 10 — Figure S10. Plasma renin levels during hypovolemia in PIEZO-deficient mice, related to Figure 7. A. Simple linear regression of matched data points (same mouse) from Figures 3A–C and 7B–D with indicated R squared value (95% C.I. of slope = 0.4478 to 0.6414). B. Simple linear regression of matched data points (same mouse) from Figures 3A–B and 7B–C with indicated R squared value (95% C.I. of slope = 33.92 to 49.82). C. Plasma renin levels in Piezo1fl/fl; Piezo2fl/fl; PdgfrbWT versus Piezo1fl/fl; Piezo2fl/fl; PdgfrbCreERT2 animals six hours following PEG injection (Mann–Whitney: *p = 0.0315, U = 16; n = 9 PdgfrbWT and 9 PdgfrbCreERT2 mice). D. Plasma renin levels in Piezo1fl/fl; Piezo2fl/fl; SNSWT versus Piezo1fl/fl; Piezo2fl/fl; SNSCre animals six hours following PEG injection (Mann–Whitney: p = 0.2614, U = 28; n = 12 SNSWT and 7 SNSCre mice). Error bars represent mean ± s.e.m. [file NIHMS2122356-supplement-10.tiff]

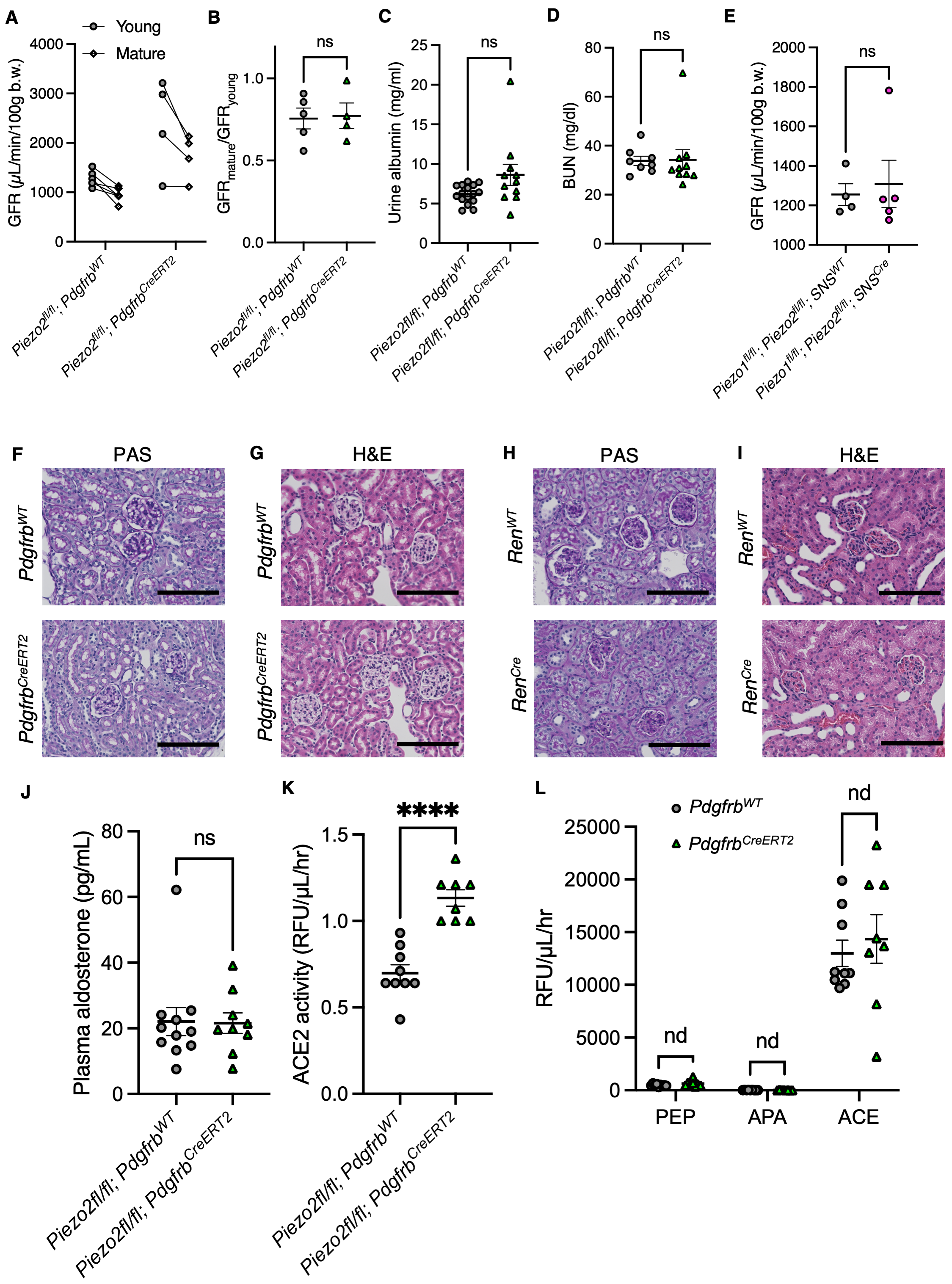

Supplement: 11 — Figure S11. PIEZO2 contributes to renin regulation independently of sympathetic and macula densa signaling, related to Figure 7. A. Experimental strategy (created with BioRender.com). B. Sectioned mouse kidney stained with anti-tyrosine hydroxylase antibody and DAPI after vehicle treatment. C. Sectioned mouse kidney with stained with anti-tyrosine hydroxylase antibody and DAPI after 6-OHDA treatment. Scale bars = 100 μm. Each experiment was repeated on N=2 mice. D. Plasma renin levels (two-way ANOVA: **pinteraction = 0.0059, F(1,29) = 8.831496; Uncorrected Fisher’s LSD (left to right): **** p < 0.0001, **** p < 0.0001, ****p < 0.0001, ***p = 0.0001; n = 8 RenWT saline, 8 RenCre saline, 9 RenWT PEG, and 8 RenCre saline mice). E. Plasma aldosterone levels in mice from D, except for 2 RenWT saline samples that were untested due to insufficient sample volume (two-way ANOVA: ***pgenotype = 0.0004, F(1,27) = 16.17869; Uncorrected Fisher’s LSD (left to right): p = 0.4419, **** p < 0.0001, ****p < 0.0001, **p = 0.0010; n = 6 RenWT saline, 8 RenCre saline, 9 RenWT PEG, and 8 RenCre saline mice). Data from D and E were subjected to a log-transform prior to statistical analysis. Each experiment was performed on at least two independent cohorts of mice, and error bars represent mean ± s.e.m. [file NIHMS2122356-supplement-11.tiff]
