# Supplementary material for: Renal PIEZO2 is an essential regulator of renin
Source: Cell. Author manuscript; Available in PMC 2025 Dec 11. (PMC12695021; doi:10.1016/j.cell.2025.11.013)
Supplement: 12 [file NIHMS2122356-supplement-12.pdf]

Mouse line	Activity	Cell type specificity	Notes
Pdgfrb^{CreERT2}	Tamoxifen induction in adults	Most mural cells including vascular pericytes, glomerular mesangial cells, JG cells, and the mural cells along the afferent and efferent arterioles; additionally targets a variety of other stromal populations including fibroblasts	For the *in vivo* experiments, the knock-in mouse line targeted to the endogenous *Pdgfrb* gene locus was used. The snRNA-seq utilized the transgenic mouse line (see Methods).
FoxD1$^{GFP\text{-}Cre}$	Constitutive	Stromal cell progenitors that give rise to fibroblasts, vascular pericytes, glomerular mesangial cells, JG cells, and the mural cells along the afferent and efferent arterioles	Targeted to the endogenous *Foxd1* locus
Ren^{Cre}	Constitutive	Cells of renin lineage including JG cells and mural cells along the afferent and efferent arterioles in uninjured kidneys. We observe that this Cre line does not target intraglomerular mesangial cells or pericytes beyond the preglomerular vessels.	This transgenic Cre line is on a *Ren1c* monogenic background.
Ren^{CreER}	Tamoxifen induction in adults	Renin-expressing cells at the time of tamoxifen injection encompassing JG cells and a smaller proportion of mural cells of renin lineage than *Ren*^{Cre}. While this line is more selective than *Ren*^{Cre} , we found it to be unsuitable for loss-of-function studies due to incomplete targeting of adult renin-expressing cells.	This transgenic Cre line is on a *Ren1c* monogenic background. It only partially recombines in renin-expressing cells, targeting only a fraction of presumptive JG cells.
SNS^{Cre}	Constitutive	80% of peripheral somatosensory and vagal neurons	This transgenic Cre line utilizes the *Scn10a* promoter (Nav1.8 ion channel)

Table S1. This table provides a description of Cre lines used in the study, related to Figures 1 and 3. See **STAR Methods** for further details on individual mouse lines.
